# Trend analysis and future projections of global burden of opioid use disorder (OUD) from 1990 to 2030

**DOI:** 10.3389/fphar.2025.1669269

**Published:** 2025-11-25

**Authors:** Wenshuo Jiang, Zhigang Zhao, Bin Zhu

**Affiliations:** 1 Department of Pharmacy, Beijing Tiantan Hospital, Capital Medical University, Beijing, China; 2 School of Pharmaceutical Sciences, Capital Medical University, Beijing, China

**Keywords:** opioid use disorder, global burden of disease study, incidence, DALYs, mortality

## Abstract

**Objectives:**

Opioid Use Disorder (OUD) is a chronic medical crisis which represents significant public health challenge on global scale. We aim to provide long-term trends and future projections of OUD for effective intervention.

**Methods:**

This study utilized data from the Global Burden of Disease (GBD) study 2021 for analysis. OUD burden was assessed using absolute numbers and age-standardized rates of incidence (ASIR), prevalence (ASPR), disability-adjusted life years (ASDR), and mortality (ASMR) per 100,000 population, with 95% uncertainty intervals (UIs). Temporal trends were analyzed using joinpoint regression. Age-period-cohort (APC) models were applied to assess the independent effects of age, time period, and birth cohort on OUD burden. Decomposition analysis quantified the relative contributions of population growth, aging, and epidemiological changes to the overall burden variation. Finally, autoregressive integrated moving average (ARIMA) models were used to forecast OUD burden through 2030.

**Results:**

In 2021, an estimated 1.94 million new cases and 16.16 million prevalent cases of OUD were recorded globally, resulting in 11.22 million DALYs and nearly 99,556 deaths. The number of incidence, prevalence, DALYs and mortality of OUD all showed substantial increases. The age-standardized rates also increased but the margins were relatively small. The highest levels and fastest growth were observed in high-SDI regions, particularly North America. Males consistently exhibited higher DALY and mortality rates than females. The burden was greatest among individuals aged 15–49 years. Joinpoint analysis revealed fluctuating trends with notable increases after 2010. APC analysis showed peak incidence at ages 20–25 and declining risk in later birth cohorts. Decomposition analysis indicated that population growth and epidemiological changes were the main contributors to the rising burden. ARIMA forecasting predicted continued increases in incidence and DALYs but slight declines in prevalence and mortality by 2030.

**Conclusion:**

The global burden of opioid use disorder (OUD) has continued to rise since 1990, mainly driven by population growth and epidemiological changes. Although age-standardized rates have remained stable or increased slightly, regional disparities persist, with the highest burden in high-SDI areas. Forecasts suggest modest increases in incidence and DALYs by 2030, underscoring the need for sustained, adaptive policies and preventive strategies to mitigate the evolving opioid crisis.

## 1 Introduction

Opioid use disorder is a recurrent chronic disorder, initiated by the activation of brain reward neurocircuits, and characterized with a strong craving for and continued use of opioid, despite impairment and distress (Strang et al., 2020; Taylor and Samet, 2022). Globally, more than 36 million people suffered from opioid dependence in 2021, and over 12 million people died from opioid related disorders annually (Degenhardt et al., 2019; Zhang et al., 2025). As a long-term disorder, drug use disorder can lead to significant neurological damage, affecting cognitive function and mental health, while also increasing the risk of cardiovascular diseases, potentially resulting in suicide, comorbid mental illness, and premature mortality (Kim et al., 2020). OUD is emerging as a global health crisis of significant scope (Krausz et al., 2021).

Prior researches suggest that the burden of OUD burden varies significantly across different regions, sexes, and ages and increases sharply in recent years (Gorfinkel et al., 2024; Han et al., 2015; Martins et al., 2017; Lin et al., 2019; Shoff et al., 2021). The demographic disparities in the development and management of OUD necessitate further evidence (Siddiqui and Urman, 2022). A comprehensive analysis is needed to provide further evidence on the temporal trends, global disparities and impact of intervention strategies on burden of OUD.

To fill this gap, we used data from 2021 GBD study to examine trends and future projections of OUD globally. The global burden of disease (GBD) research is a comprehensive and influential public health research initiatives worldwide for appreciating the epidemiological landscape of different illnesses. Previous researches on OUD utilizing GBD data (Orpana et al., 2018; Rajkumar, 2021; Han et al., 2025; Fang et al., 2025) demonstrated the time trends and disparities by age, sex and region. However there lacks evidence on the changing points, contributing factors and future predictions. Therefore, we aimed to provide more detailed, comprehesive trend analysis, make numerical forecasts by 2030 and reflect the possible impact of factors such as aging, policy implementation and epidemiological changes on the variation of burden of OUD (Fang et al., 2025).

## 2 Materials and methods

### 2.1 Study design and data sources

The data used in this study was extracted from the Global Burden of Disease (GBD) 2021 dataset (IHME http://ghdx.healthdata.org/gbd-results-tool), which provides comprehensive estimates of disease burden for over 300 diseases and injuries across 204 countries and territories (Naghavi et al., 2024). The dataset utilizes a robust methodology, integrating data from various sources such as vital registration systems, health surveys, and disease registries, along with statistical modeling to fill in gaps (GBD, 2024). Given that the 2021 GBD data is publicly available, the institutional ethics committee approved a waiver for this study, as no ethical approval was required. This study adhered to the guidelines for accurate and transparent health assessment reporting (Castaldelli-Maia et al., 2023).

### 2.2 Outcome measures

In the GBD 2021, the opioid use disorder is defined as a chronic, relapsing condition characterized by a pattern of problematic opioid use that leads to significant impairment or distress, according to the ICD-10 codes of F11.0-F11.9, P96.1, and R78.1 and DSM-IV (Diagnostic and Statistical Manual of Mental Disorders, Fourth Edition) code of 304.00 (Effatpanah et al., 2025). The GBD quantifies OUD as part of the broader category of “substance use disorders.”

In this study, we used four measures including the incidence, prevalence, mortality, and disability-adjusted life years (DALYs) to reflect the burden of OUD. Incidence is the number of new OUD cases per 100,000 population annually. Prevalence is the total number of existing OUD cases per 100,000 population annually. DALYs combines years of life lost to premature death and years lived with disability (Degenhardt et al., 2018; Zhang et al., 2024). Mortality is the annual number of deaths due to OUD per 100,000 population. The age-standardized indicators used in this study include ASIR (Age-Standardized Incidence Rate), ASPR (Age-Standardized Prevalence Rate), ASDR (Age-Standardized DALY Rate) and ASMR (Age-Standardized Mortality Rate). The UIs (Uncertainty Intervals) were derived using the Global Burden of Disease (GBD) study’s method of 1,000 ordered draws, which strengthens both the accuracy of the estimates and the reliability of the overall findings.

### 2.3 Statistical analysis

DisMod-MR (Disease Model with Multiple Causes of Death and Comorbidities) and CODEm (Covariate-Adjusted Death and Disability Estimation Model) were major tools used in the study to estimate health metrics (GBD 2021 Di sease et al., 2024). We computed the estimated annual percentage change (EAPC) using a least squares linear regression model, and the regression outcomes were systematically structured and analyzed employing the broom package26. The EAPC is calculated by: ln(y) = α + βx + ε, where (y) signifies the age-standardized incidence, (α) is the intercept, (x) represents the year, (β) is the slope, and (ε) is the normally distributed error term. The 95% CI of the EAPC was derived from the standard error of β in the regression model, using the formula: 95% CI = 100 × [exp(β ± 1.96 × SE(β)) − 1].

We created global maps and performed regional comparison analysis to examine the worldwide distribution and regional variations in OUD. Data were compiled based on the geographic areas established by the GBD research (Feigin et al., 2024). Data were categorized to standardized 5-year age groups for both men and women. The SDI categorization (low, low-middle, medium, high-middle, and high) was used to compare illness burden across varying degrees of socioeconomic development (Zi et al., 2024).

#### 2.3.1 Joinpoint regression model analysis

The identification of temporal trends were carried out by joinpoint regression model. The joinpoint regression model, first introduced by Kim et al. (2000), identifies significant change points in trend data by fitting linear segments to time series data. In this study, we carried out Joinpoint analysis in the joinpoint regression 4.9 software (Statistical Research and Applications Branch, National Cancer Institute, United States of America). We calculated the best-fitting lines using permutation tests, with overall significant level set at 0.05. The key turning points were identified where statistically significant changes in slope occur. We also calculated the Average Annual Percent Change (AAPC), and the 95% confidence interval (CI) was determined using parametric method.

#### 2.3.2 Age-period-cohort (APC) model analysis

Age‐period‐cohort (APC) models were applied to analyze the influence of age, time period, and birth cohort on OUD burden (Rosenberg, 2019). Because age, period, and cohort are linearly dependent (cohort = period–age), conventional regression models face a perfect collinearity problem. The APC model uses a intrinsic estimator approach to overcome this and yield unique and unbiased estimates of the three effects while maintaining orthogonality among predictors. In this study, age-period-cohort model was created in the National Cancer Institute APC (NCI https://analysistools.cancer.gov/apc/) modelling analysis tool. Age and period were first divided into 5-year continuous intervals from 15–20 to 95+, and from 1992–1997 to 2017–2021, respectively. Twenty-two birth cohorts were summarized from 1895–1900 to 2000–2005. The intrinsic estimator (IE) method was integrated into the age-period-cohort model to estimate the net effects for three dimensions (Zou et al., 2022). The reference categories were set as the age of 40–45, the period of 2001–2006, and the birth cohort of 1946–1951 to ensure interpretability and model stability. The relative risk (RR) value for each age, period, and cohort represents the independent risk compared to the reference group (Holford, 1991).

#### 2.3.3 Decomposition analysis

To identify the main contributors to changes in the OUD burden, we performed a decomposition analysis based on the Global Burden of Disease (GBD) 2021 estimates. This approach quantifies the relative contributions of population growth, population aging, and changes in age-specific rates to the overall variation in incidence, prevalence, DALYs, and mortality between two time points. Following the GBD analytical framework, the total change in disease burden was decomposed using a stepwise replacement method, allowing isolation of the net effect of demographic and epidemiological factors. Results were expressed as percentage contributions of each component to the total change.

#### 2.3.4 Autoregressive integrated moving average (ARIMA) model analysis

Autoregressive integrated moving average model (ARIMA) was used to forecast the burden of OUD. The ARIMA model is denoted as ARIMA(p,d,q), where p denotes the number of lag observations (AR term), d represents the degree of differencing (I term), and q symbols the size of the moving average window (MA term) (Luo, 2013). To carry out the analysis of ARIMA models, the stationarity of the time series was examined using time series plots. For non-stationary series, differencing was applied until stationarity was achieved. The autocorrelation function (ACF) and partial autocorrelation function (PACF) plots were analyzed to determine the appropriate values of *p*, *q*, and *d*. The optimal model was selected based on the Akaike Information Criterion (AIC) (Martins et al., 2019). Residuals of the selected model were further tested using the Ljung–Box test, with the significant level set at 0.05. The optimal model was then used to perform forecasts on the time series data.

## 3 Results

### 3.1 Description of OUD burden

#### 3.1.1 Incidence

Globally, there were 1,942,525 new cases (95% confidence interval [CI]: 1,643,342–2,328,363) of OUD in 2021 (51.75% males). The ASIR was 24.5 (95% confidence interval [CI]: 20.7–29.5) per 100,000 population. The EAPC of incidence was 1.03 (95% confidence interval [CI]: 0.88–1.18). Among the 5 SDI regions, the high SDI region showed the highest incidence for OUD of 1942525.3 (95% confidence interval [CI]: 1643342.3–2328363.2). The EAPCs were all positive in high, low-middle, and low SDI regions, but negative in high-middle and middle SDI regions. The EAPC of the high SDI region were the highest. Among the 21 regions, North America had the highest incidence of 456336.9 (95% confidence interval [CI]: 382679.8–549886.4) Moreover, high-income North America also showed highest EAPCs of incidence (Table 1).

**TABLE 1:** All-age cases, age-standardized rates and estimated annual percentage change (EAPC) of incidence for opioid use disorder (OUD).

Location	1990		2021		
	Absolute numbers	Age-standardized rate (per 100.000)	Absolute numbers	Age-standardized rate (per 100.000)	EAPCs
Global	1301550.8 (1077634.5, 1598052.6)	23.4 (19.6, 28.5)	1942525.3 (1643342.3, 2328363.2)	24.5 (20.7, 29.5)	−0.17 (−0.34, 0)
High SDI	207664.2 (173326.4, 252998.6)	22.8 (19, 27.8)	609680.5 (518566.1, 721842.4)	68.5 (57.7, 82.3)	3.66 (3.23, 4.1)
High-middle SDI	390707.1 (326323.4, 477149.1)	33.7 (28.2, 41)	335809.9 (284830.5, 398898.7)	27.2 (23, 32.6)	−1.36 (−1.78, −0.95)
Middle SDI	450750.7 (371017.8, 556894.1)	24.4 (20.5, 29.6)	469928.3 (393636.6, 562644.1)	18.8 (15.8, 22.8)	−1.25 (−1.43, −1.08)
Low-middle SDI	190951.3 (152928.2, 241535.1)	17.1 (14.1, 21)	370910.6 (305808.9, 455797.9)	18.2 (15.3, 22.2)	0.09 (−0.01, 0.18)
Low SDI	60715.1 (48591.6, 77000.6)	13.7 (11.3, 16.8)	155169.7 (124662.8, 194320.3)	14.4 (11.9, 17.4)	0.1 (0.06, 0.14)
Andean Latin America	6714.5 (5158.3, 8555.1)	17 (13.2, 21.4)	12379.6 (9822.7, 15470.7)	17.4 (13.8, 21.7)	0.14 (0.06, 0.22)
Australasia	9220.1 (7965.2, 10661.8)	43.4 (37.6, 50)	12977.3 (11088.9, 15009.4)	44.9 (38.7, 52)	−0.34 (−0.76, 0.07)
Caribbean	6947.8 (5502.8, 8823.1)	18.1 (14.4, 22.3)	7626.9 (6095.7, 9436.5)	15.6 (12.5, 19.3)	−0.59 (−0.67, −0.51)
Central Asia	25909.3 (20775.1, 31992.2)	36.3 (29.7, 44.4)	35075.2 (29584.4, 41820)	36.7 (31, 43.7)	−0.07 (−0.22, 0.09)
Central Europe	16514 (13678.3, 19961.7)	13.3 (10.8, 16.2)	16499.1 (14140.6, 19429.5)	16.1 (13.6, 19)	0.55 (0.42, 0.67)
Central Latin America	27556.9 (21406.1, 35169.8)	16.1 (12.8, 20.1)	40991.3 (32758.1, 50724.7)	15.2 (12.2, 18.9)	−0.17 (−0.26, −0.07)
Central Sub-Saharan Africa	5588.6 (4362.1, 7057.1)	11.4 (9.2, 14.1)	16045.2 (12747.9, 20234.1)	12.5 (10.2, 15.4)	0.38 (0.35, 0.42)
East Asia	419882.2 (348058.6, 515521.1)	30.3 (25.6, 36.4)	244997.9 (202875.9, 293576.9)	16.7 (13.9, 20.3)	−2.82 (−3.17, −2.47)
Eastern Europe	152973.5 (127089.3, 183616.1)	69.6 (57.8, 83.8)	129174.8 (110164, 153540.4)	73.3 (61.9, 87.3)	−0.57 (−1.22, 0.07)
Eastern Sub-Saharan Africa	17387 (13745.4, 22221)	10.7 (8.8, 13.2)	42755 (34331.9, 53621.4)	10.8 (8.9, 13)	−0.05 (−0.09, −0.02)
High-income Asia Pacific	27979.6 (22475.6, 34511.6)	15.1 (12.1, 18.9)	23884.5 (19434.4, 28719.2)	14.9 (12, 18.4)	−0.02 (−0.13, 0.08)
High-income North America	86864.4 (71332, 108021.1)	30.2 (24.8, 37.7)	456336.9 (382679.8, 549886.4)	144.2 (120.1, 174.9)	5.72 (5.1, 6.34)
North Africa and Middle East	121489.9 (94834.3, 154339.6)	34.8 (28.1, 43)	245270.7 (203638.3, 296380.1)	37.8 (31.5, 45.6)	0.3 (0.14, 0.47)
Oceania	767.3 (608.3, 972.6)	12.1 (9.9, 14.8)	1737.4 (1407, 2154.9)	12.5 (10.2, 15.2)	0.11 (0.1, 0.12)
South Asia	175002.5 (140845.6, 221256.6)	16.7 (13.8, 20.7)	378428.5 (309714.6, 466046.2)	18.9 (15.7, 23.1)	0.13 (−0.1, 0.36)
Southeast Asia	43497.8 (34656.2, 53758.6)	9.2 (7.6, 11.2)	68526.4 (56760.6, 82426.8)	9.4 (7.8, 11.2)	−0.08 (−0.16, 0.01)
Southern Latin America	8959.7 (6918.5, 11183.6)	17.9 (13.9, 22.3)	12389.4 (9945.7, 15253)	17.8 (14.1, 22.1)	−0.12 (−0.2, −0.03)
Southern Sub-Saharan Africa	17179.6 (14051.5, 21388.5)	32.9 (27.4, 39.8)	19234.1 (16063.8, 23406)	23.3 (19.7, 28.1)	−1.44 (−1.77, −1.1)
Tropical Latin America	27856.9 (21477.5, 35429.5)	17.1 (13.4, 21.5)	37570.4 (29895.5, 46843.2)	15.8 (12.4, 19.7)	−0.22 (−0.34, −0.1)
Western Europe	84152.9 (71514.3, 99715.3)	21.6 (18.3, 25.7)	90781.8 (78907.1, 104670.7)	24.1 (20.7, 28.1)	−0.27 (−0.59, 0.05)
Western Sub-Saharan Africa	19106.2 (15023.5, 24339.9)	11 (8.9, 13.6)	49843.1 (39458.8, 63152.5)	10.9 (9, 13.3)	−0.03 (−0.07, 0.01)

#### 3.1.2 Prevalence

The number of existing OUD cases is 16,164,876 (95% confidence interval [UI]: 14,133,120–18,431,510) worldwide in 2021 (50.59% males). The ASPR was 198.5 (95% confidence interval [CI]: 173.4–227.2) per 100,000 population. The EAPC of prevalence was −0.17 (95% confidence interval [CI]: −0.34–0). Among the 5 SDI regions, the high SDI region showed the highest prevalence for OUD of 8316982.4 (95% confidence interval [CI]: 7416372.9–9351503.5). Among the 21 regions, North America had the highest prevalence of 6894161.3 (95% confidence interval [CI]: 6086133.8–7821275) (Supplementary Table S1).

#### 3.1.3 DALYs

The OUD resulted in 11218519 DALYs (95% confidence interval [UI]: 9188657.5–13159551.4) totally in 2021, with males accounting for 6745373 years (60.13%). The ASDR was 137.2 (95% confidence interval [CI]: 112.3–161.4). The EAPC of DALYs was 0.5 (95% confidence interval [CI]: 0.3–0.71). Among the 5 SDI regions, the high SDI region showed the highest DALYs of 6548594.7 (95% confidence interval [CI]: 5421925.8–7567176.6). Among the 21 regions, North America had the highest mortality of 58205.5 (95% confidence interval [CI]: 51549–65872.2) (Supplementary Table S2).

#### 3.1.4 Mortality

There were a total of 99,556 death cases (95% confidence interval [CI]: 92,948–108,050) resulted from OUD in 2021(70.87% males). The ASMR was 1.19 (95% confidence interval [CI]: 1.12–1.29). The EAPC of mortality was 0.86 (95% confidence interval [CI]: 0.68–1.04). Among the 5 SDI regions, the high SDI region showed the highest mortality of 67688.9 (95% confidence interval [CI]: 61163.6–75435.1). Among the 21 regions, North America had the highest mortality of 58205.5 (95% confidence interval [CI]: 51549–65872.2) (Supplementary Table S3).

#### 3.1.5 Heatmaps of OUD burden

**FIGURE 1:**
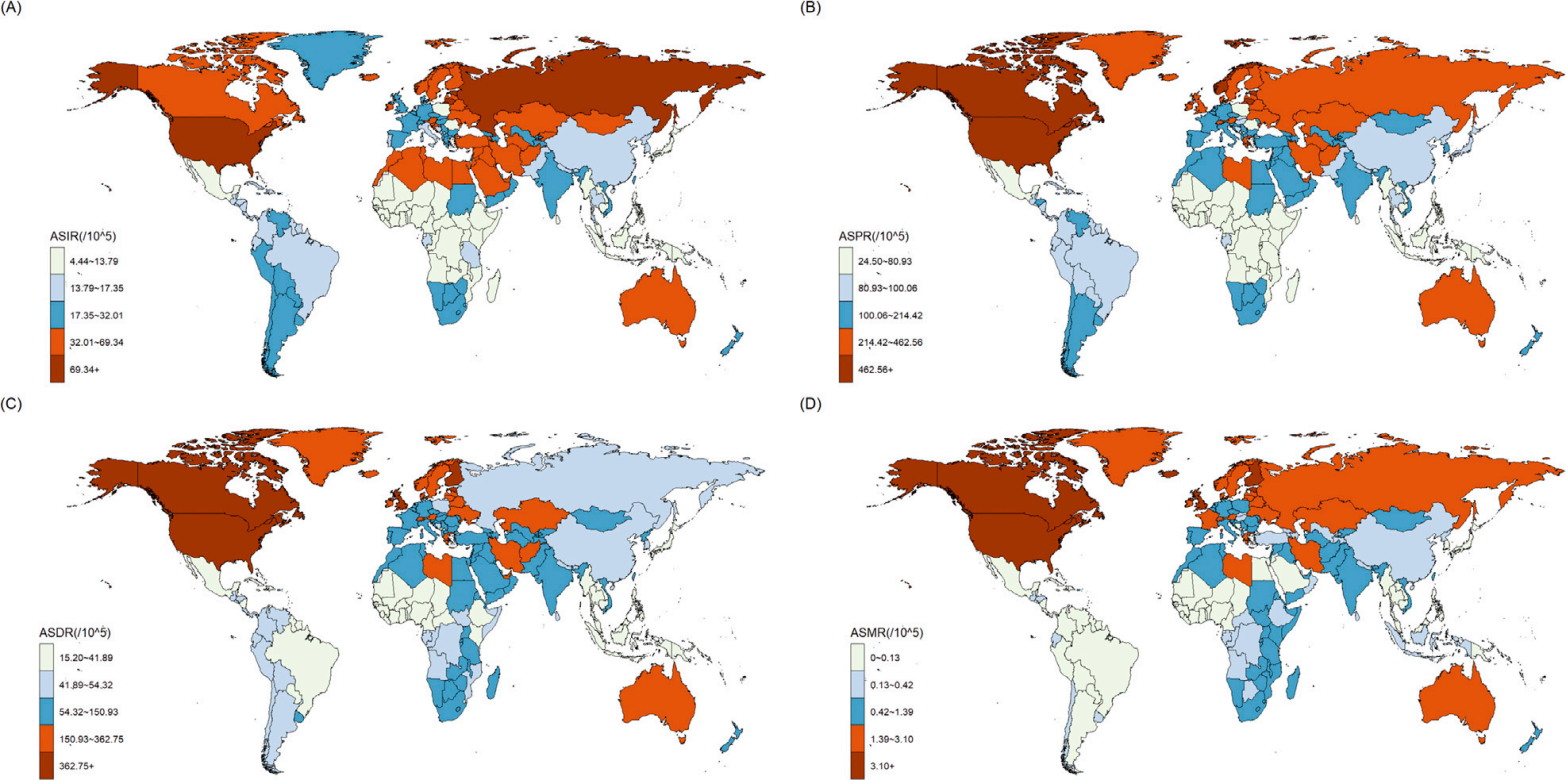
The distribution of age-standardized rates (ASR) in the GBD 204 countries and regions: **(A)** age-standardized incidence rate (ASIR); **(B)** age-standardized prevalence rate (ASPR); **(C)** age-standardized DALYs rate (ASDR); **(D)** age-standardized mortality rate (ASMR).

**FIGURE 2:**
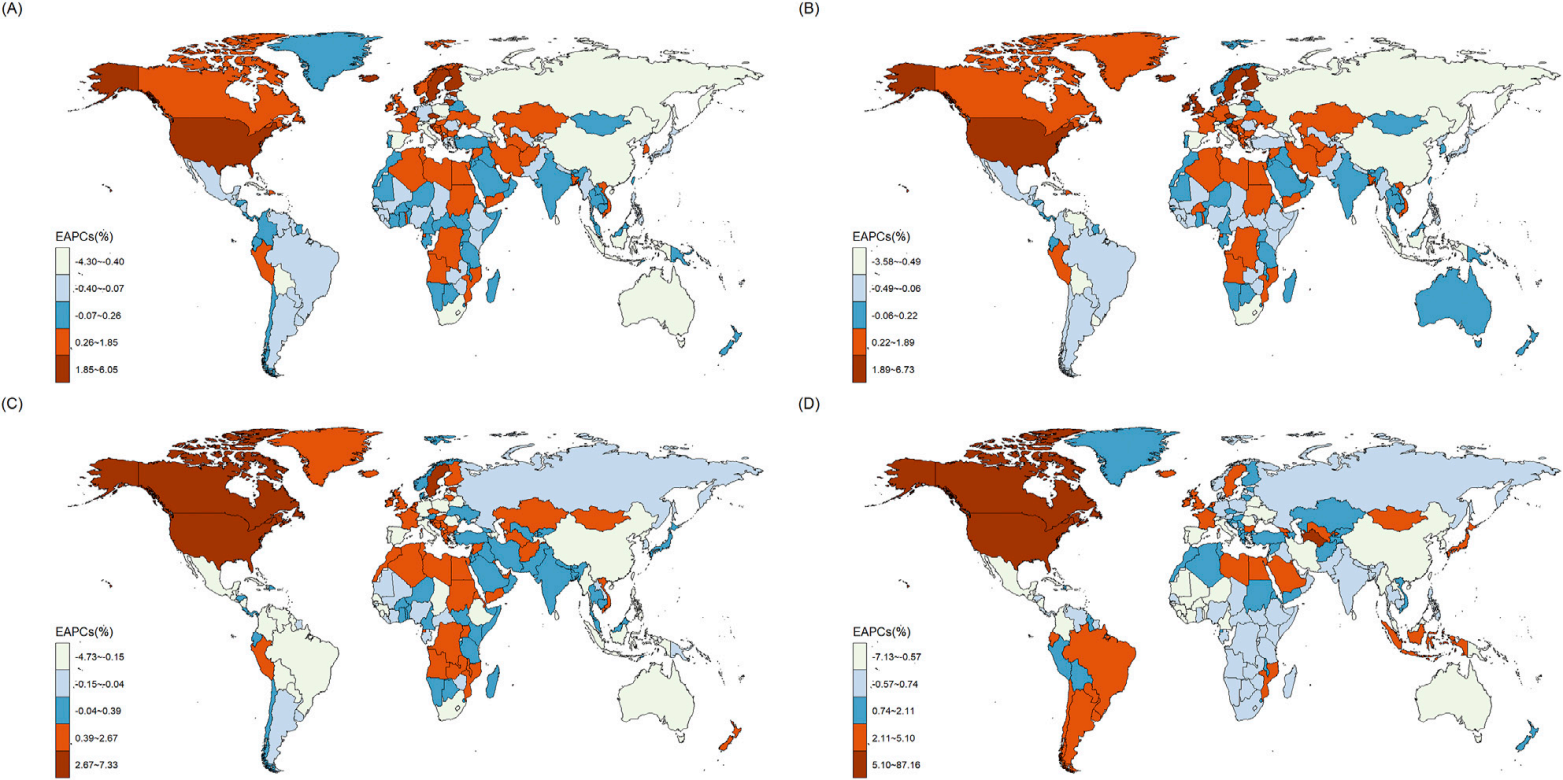
The Estimated Annual Percentage Change (EAPC) in the GBD 204 countries and regions: **(A)** age-standardized incidence rate (ASIR); **(B)** age-standardized prevalence rate (ASPR); **(C)** age-standardized DALYs rate (ASDR); **(D)** age-standardized mortality rate (ASMR).

Figure 1 showed the heatmap of the OUD burden including ASIR, ASPR, ASDR and ASMR in 204 countries and regions in 2021. High rates of OUD burden were mostly distributed in North America, Oceania and Europe. Figure 2 showed the heatmap of EAPCs of OUD burden globally in 2021. The North America showed the highest EAPCs for ASIR, ASPR, ASMR and ASDR. Other regions with high EAPCs located mostly in Africa. The South America showed significantly high EAPCs in ASMR.

### 3.2 Distribution across age, sex and period

**FIGURE 3:**
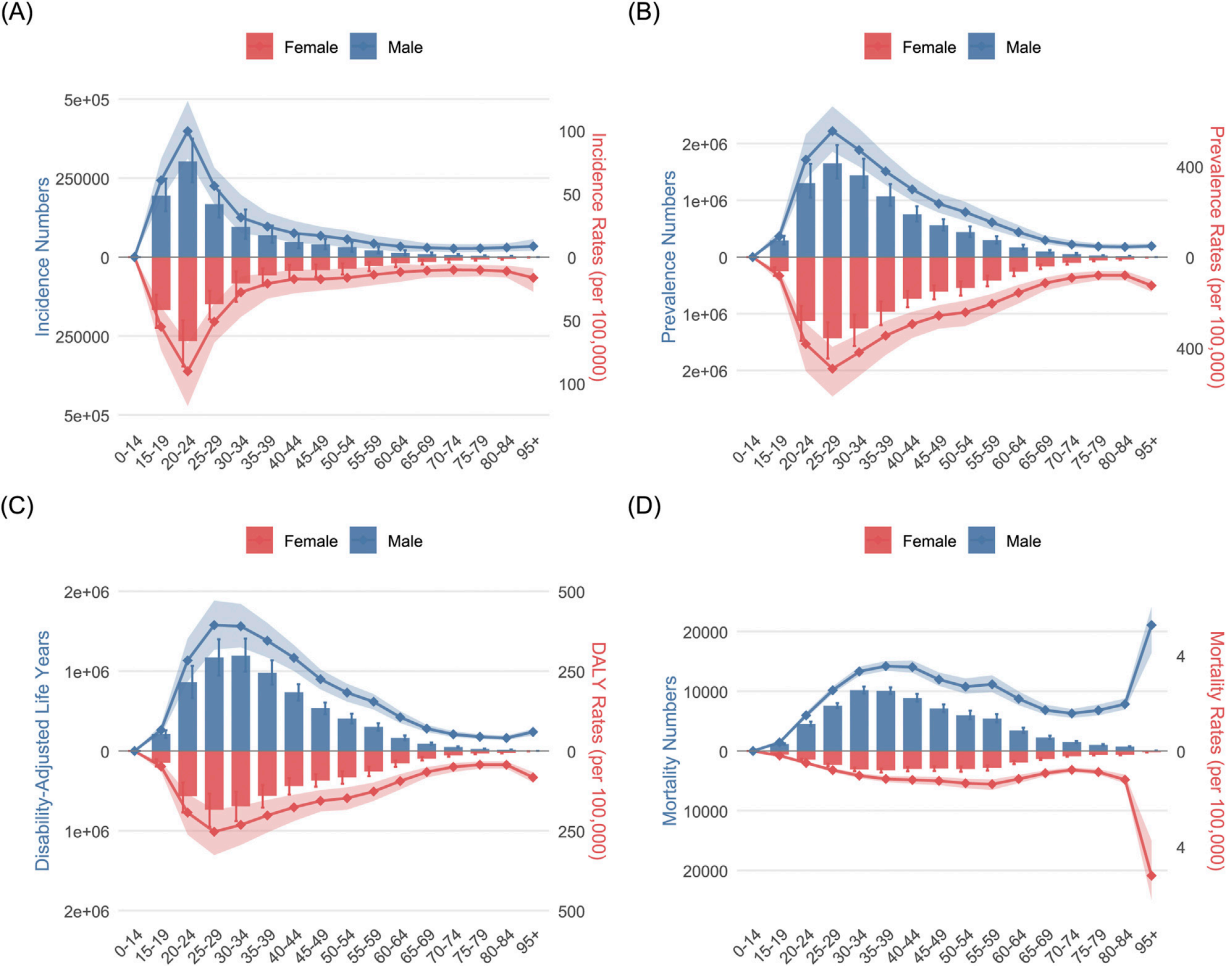
The age-sex correlation analysis results of opioid use disorder (OUD) burden: **(A)** incidence; **(B)** prevalence; **(C)**; DALYs; **(D)** mortality.

The examination of age-sex correlations in 2021 reveals that the incidence of OUD escalates from 0 to 24 years old, and then gradually declines after the age of 24 (Figure 3A). The prevalence of OUD increases significantly under 29 years old, reaching a zenith at around 25–29 years before seeing decline (Figure 3B). DALYs also increase significantly with age before 29 years and steadily decrease after 29 years (Figure 3C). The mortality worldwide showed increasing trends between 0 and 39 years, and then descend in a winding manner. The age-standardized mortality saw a surge above 80 years old (Figure 3D). These tendencies were uniform across genders. But the DALYs and mortality of male were significantly higher than female.

The age-time correlation study indicates the burden in population aged between 15 and 49 years were the heaviest and there was a steady increase in OUD incidence among those 15–49 years old worldwide and across high, low-middle and low SDI areas. While in high-middle and middle SDI areas, the incidence and prevalence rise before around 2005 years and decrease afterward with a little rise in recent years among individuals of 15–49 years whereas the DALYs and mortality had similar tendency but changed in a winding manner (Supplementary Figures S1–S4).

The examination of sex-time correlations indicates a progressive increase in the worldwide burden of OUD across all gender demographics. The high, low-middle and low SDI zone experienced persistent rise in OUD burden, while in the high-middle and middle SDI region, there are rise before around the 2005 years and decrease after that with a little rise in recent years, including both males and females (Supplementary Figures S5–S8).

### 3.3 Correlation between SDI and OUD burden

The connection between the SDI and the ASIR, ASPR and ASDR of OUD was nonlinear both globally and across the 21 GBD areas. The OUD burden was constant when the SDI was less than 0.8. However, a rise in the OUD burden with SDI was seen beyond an SDI of 0.8. While other areas showed more moderate rises, the high-income North America showed the largest increase in OUD burden (Supplementary Figure S9). A similar nonlinear association was seen across the 204 nations between SDI and the age-standardized incidence, prevalence, DALYs and mortality of OUD (Supplementary Figure S10). Countries with an SDI of more than 0.75 saw a higher OUD burden when SDI increased.

### 3.4 OUD joinpoint regression analysis results

**FIGURE 4:**
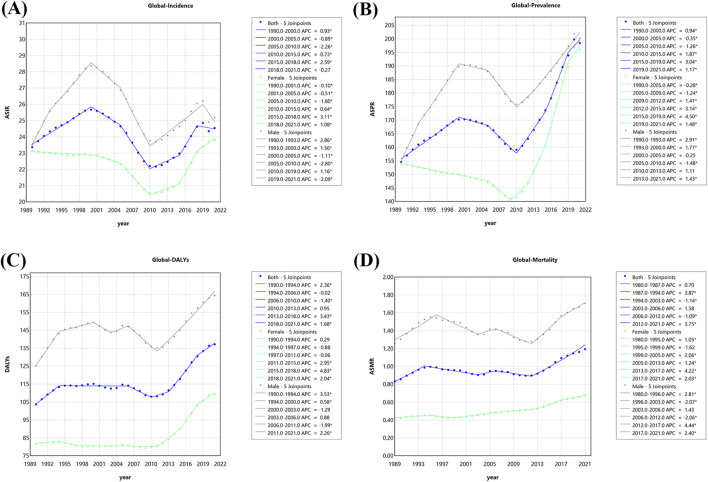
The Joinpoint analysis results of opioid use disorder (OUD) burden: **(A)** Incidence; **(B)** Prevalence; **(C)**; DALYs; **(D)** Mortality.

The temporal trends of incidence, prevalence, DALY, and mortality rates of OUD from 1990 to 2021 were analyzed using joinpoint regression (Figures 4A–D). Globally, the incidence rate of OUD exhibited a steady increase from 1990 to 2000. From 2000 to 2010, we observed a decreasing trend in incidence, and the most significant decline was seen in 2005–2010, followed by a significant rise until approximately 2019. Three key joinpoints were identified in 2000, 2005, 2010, and 2019. The trends were different between male and female. Female showed a significant decrease from 1990 to 2000, and increase from 2018 to 2021, which was adverse in male. Similarly, the prevalence rate of OUD increased from 1990 to 2000, decreased from 2000 to 2010, and significantly rise from 2010 to 2021. In contrast, the DALY and mortality rate showed a biphasic pattern, the DALY rates of OUD were relatively stable during 1990–2010, followed by a marked increase after 2010. The mortality rate displayed a similar trajectory, remaining low and stable before 2010, then rising significantly, especially among males. The results of the APCs and AAPCs were summarized in Supplementary Table S4.

### 3.5 OUD age-period-cohort analysis results

**FIGURE 5:**
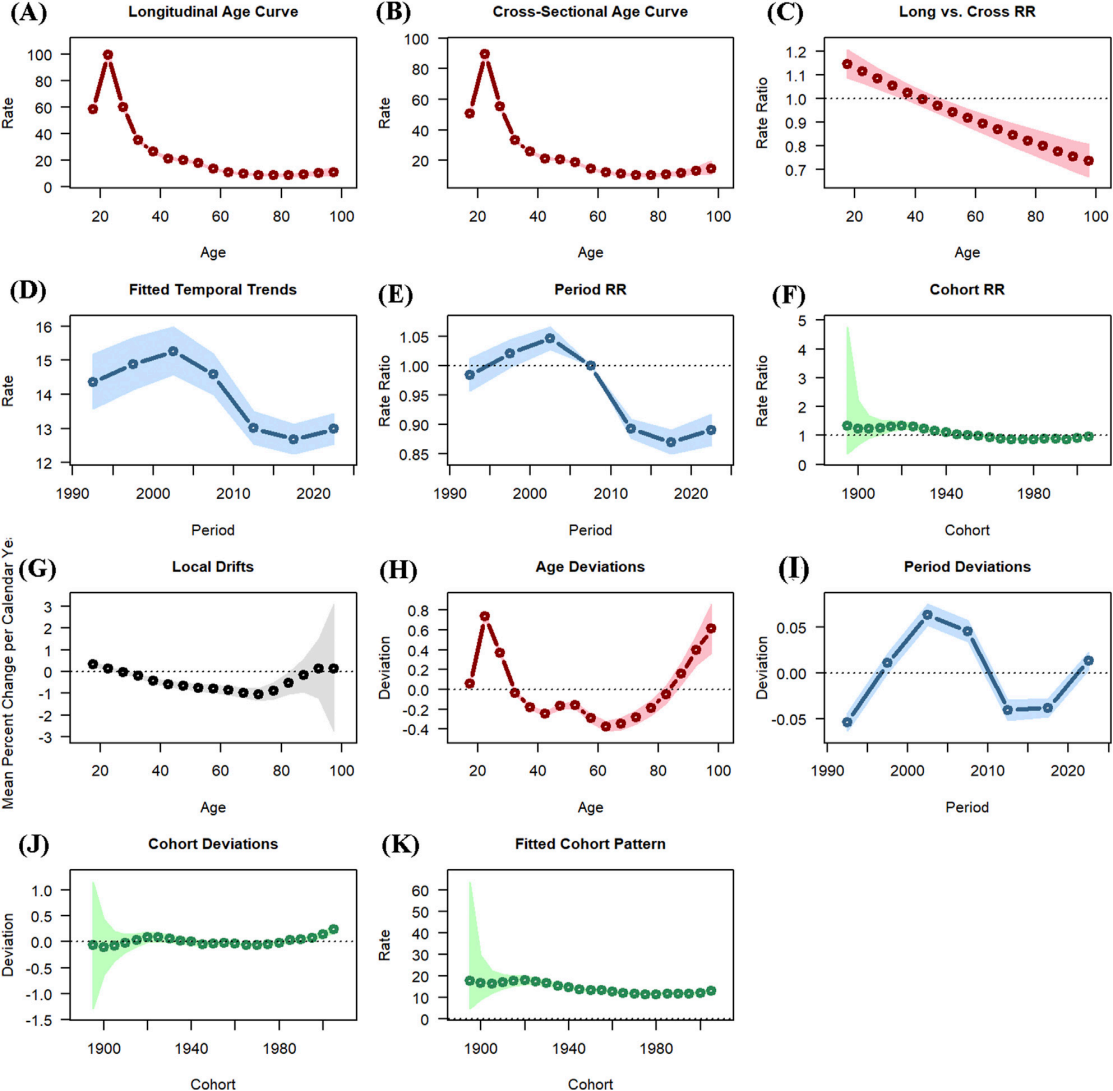
The age-period-cohort (APC) analysis of opioid use disorder (OUD) incidence: **(A)** Longitudinal age curve; **(B)** Cross-sectional age curve; **(C)** Long vs. cross RR curve; **(D)** Fitted temporal trends curve; **(E)** Period RR curve; **(F)** Cohort RR curve; **(G)** Local drifts curve; **(H)** Age deviations curve; **(I)** Period deviations curve; **(J)** Cohort deviations curve; **(K)** Fitted cohort pattern curve.

Figure 5 illustrated the age-period-cohort effect of OUD incidence. Globally, the incidence rates rose sharply between age 15 and 20, peaking at age 20–25 (Rate: 108.572, 95% CI: 103.955–113.394). A progressive decline was observed with advancing age after 20–25 years old. With regard to the period effect, the risk ratio plot demonstrated an upward trend, reaching a peak in the period group of 1997–2002 (RR: 1.044, 95% CI: 1.026–1.063), followed by a decline. The lowest risk ratio was seen in the 2011–2016 period group (RR: 0.874, 95% CI: 0.854–0.895). Regarding the cohort effect, the rask ratio plot showed a small fluctuation, increasing before the cohort group of 1916–1921 and then steadily decreasing, The lowest risk ratio was shown at group of 1991–1996. Supplementary Tables S5 and S6 provides the specific results and the Wald test statistics for the age-period-cohort model.

### 3.6 OUD decomposition analysis results

**FIGURE 6:**
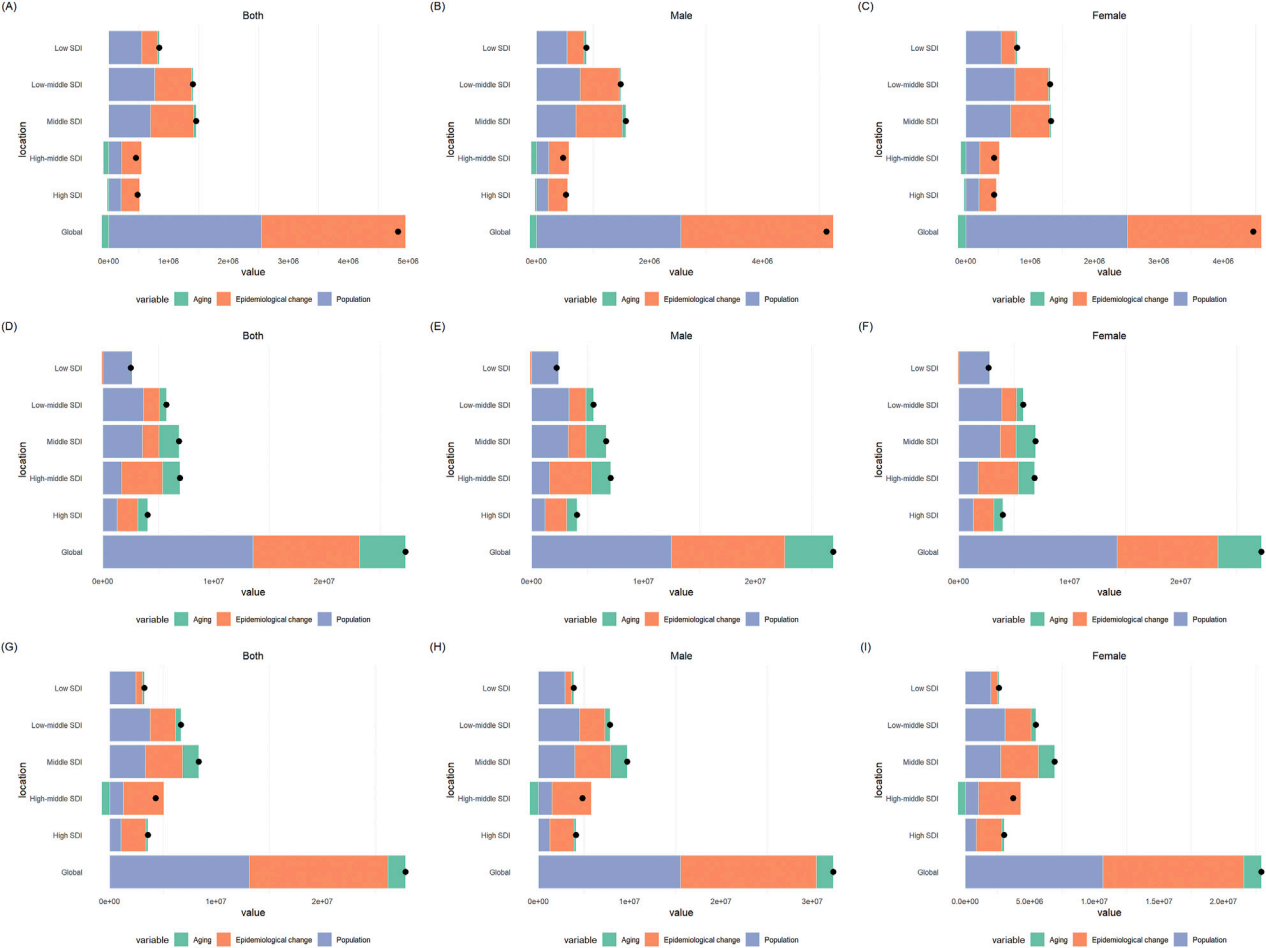
The decomposition analysis results of opioid use disorder (OUD) burden: **(A)** incidence in both sexes; **(B)** incidence in males; **(C)** incidence in females; **(D)** prevalence in both sexes; **(E)** prevalence in males; **(F)** prevalence in females; **(G)** DALYs in both sexes; **(H)** DALYs in males; **(I)** DALYs in females.

This study evaluated the impacts of aging, population and epidemiological changes on incidence, prevalence and DALYs of OUD from 1990 to 2021 (Figure 6; Supplementary Tables S7–S9). Population contributed the most to the change of incidence globally and in the 5 SDI regions, accounting for 77.26%, 74.65%, 140.09%, 78.14%, 76.3% and 73.8% respectively. Aging played a negative role in the change of incidence globally and 4 SDI regions, except for low SDI region. Regarding the change of prevalence, the contributions varied by SDI regions. Epidemiological changes contributed the most in high SDI (56.18%) and high-middle SDI regions (71.51%) while population contributed most globally (52.49%) and in middle SDI (47.38%), low-middle SDI (62.55%) and low SDI (80.89%) regions. As for the changes of DALYs, the epidemiological changes contributed the most, accounting for 58.09% globally, 74.46% in high SDI region, 105.39% in high-middle SDI region, 55.43% in middle SDI region, 47.25% in low-middle SDI region. However, the population contributed the most to the changes of DALYs in low SDI region, accounting for 55.03%.

### 3.7 OUD forecast analysis results

**FIGURE 7:**
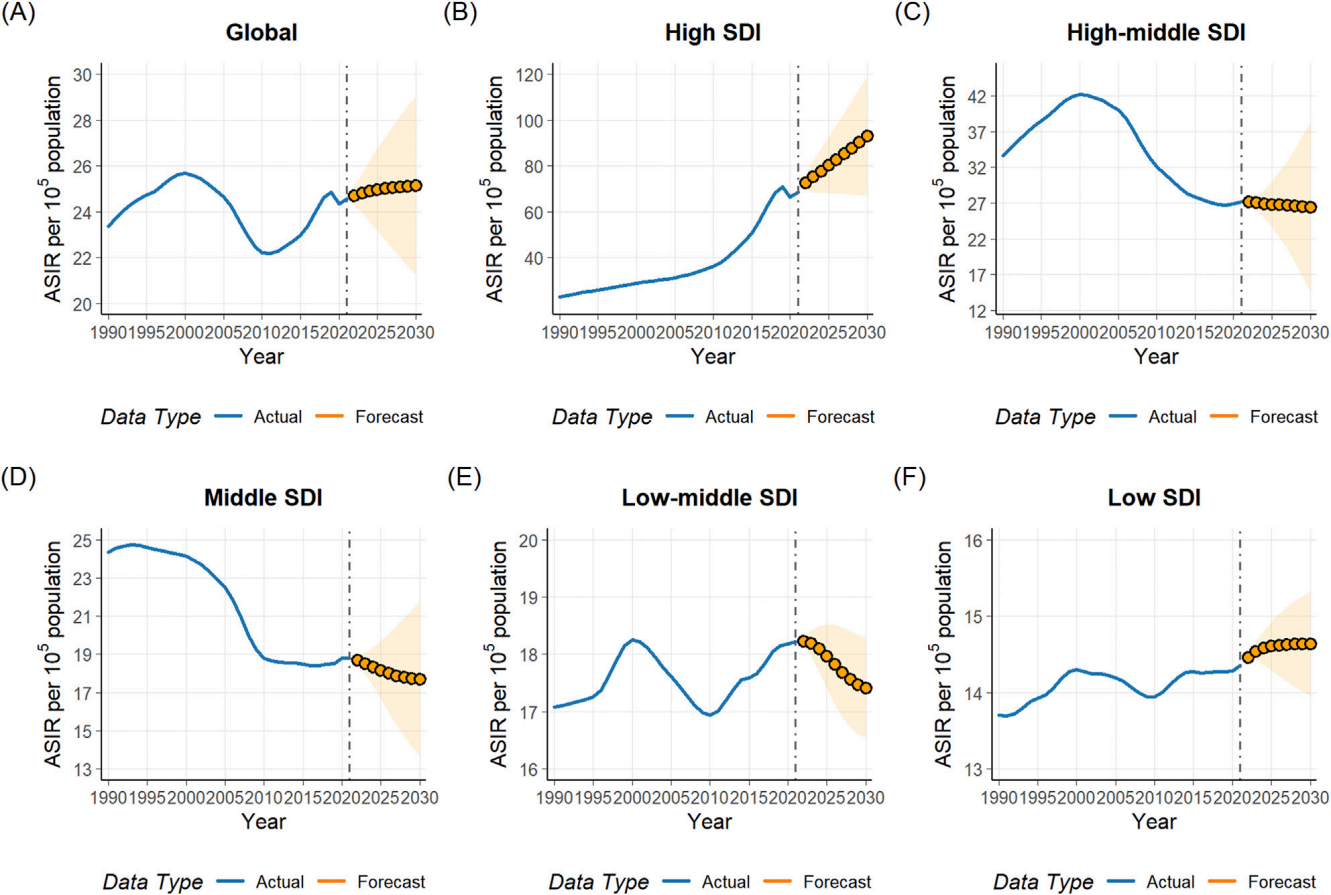
Predicted trend of age-standardized incidence rate (ASIR) from 1990 to 2030 for opioid use disorder (OUD): **(A)** global; **(B)** high SDI region; **(C)** high-middle Region; **(D)** middle region; **(E)** low-middle region; **(F)** low SDI region.

The trends of burden of OUD in the next 9 years were depicted by ARIMA models (Figure 7; Supplementary Figures S11–S13). The parameters of the optimal models selected for analysis were shown in Supplementary Table S10. Q-Q PLOTs, ACF and PACF plots of the residual errors were normally distributed as shown in Supplementary Figures S14–S25. The Ljung-Box tests confirmed the residuals of the models were white noise (Supplementary Table S11). The forecast results of burden of OUD were shown in Supplementary Table S13. Globally, the ASIR and ASDR showed increased trend, and they are predicted to increase to 25.14 (95% CI: 21.26–29.02) and 145.81 (95% CI: 128.02–163.61) per 100,000 population by 2030. On the contrast, the ASPR and ASMR demonstrated a decreasing trend and would decrease to 193.20 (95% CI: 162.20–224.20) and 1.03 (0.71–1.36) per 100,000 population by 2030.

## 4 Discussion

This study provided a systematic analysis of complex temporal changes from 1990 to 2021. We found that the number of incidence, prevalence, DALYs and mortality of OUD all showed substantial increases. However the age-standardized rates showed little increase or declined. The joinpoint analysis and APC models confirmed these findings, with the APC model exhibiting a declining tendency for period effect, and the joinpoint analysis showing a consistently decreasing trend in 2000–2010. The ARIMA model forecast that the ASIR and ASDR would increase slightly and the ASPR and ASMR would decrease by 2030.

The decomposition analysis found that the population and epidemiological changes contributed mostly for the increase in incidence and prevalence number. This may account for the substantial increases in number of cases but little variation or decline in age-standardized rates. Moreover, temporal factors, such as policy interventions or public health measures, might have contributed to the reduction in incidence risk over time. The joinpoint analysis identified a turning point around 2000, which may reflect the combined influence of policy interventions, changes in prescription practices, and public awareness. During the early 2000s, the implementation of Prescription Drug Monitoring Programs (PDMPs), stricter opioid prescribing regulations collectively reduced the medical availability and misuse of prescription opioids, leading to a temporary decline in the incidence of OUD (Rhodes et al., 2019; Furlan et al., 2014; Egan et al., 2010). After 2010, the global burden of drug use disorders increased again, which might be linked to the transition from prescription opioids to illicit opioids, especially in high-income countries (Rhodes et al., 2019; Furlan et al., 2014; Egan et al., 2010). This shift underscores the dynamic nature of the opioid epidemic and highlights the importance of sustained, adaptive control measures. From 2019, the burden of OUD decreased globally. The reason may be that the COVID-19 pandemic impeded the recording of the cases.

Consistent with previous research results, we found that OUD burden was significantly higher in population aged between 20 and 49. The APC model analyzed the age effect after excluding the effects of period and cohort, and found that the 20–25 aged population had the highest risk of developing OUD. This demographic often experiences economic instability due to transitions in education and employment (low income and job fluctuations) (Huang et al., 2011), which may exacerbate dependence on opioid. Additionally, their treatment-seeking rates are relatively low due to stigma, fear of rejection, and denial of problems (Crapanzano et al., 2019). OUD during adolescence not only increases the risk of subsequent addiction and other medical or mental health issues but may also negatively impact the developing brain in the long term (Bava et al., 2009). Therefore, adolescence is a critical period for interventions aimed at preventing severe addiction and its consequences. In the perspective of gender, burden of OUD in male were significantly higher than that in female, which are also consistent with previous studies (Orpana et al., 2018; Zhang et al., 2024). This may be due to gender-related differences in pharmacology of opioid. Opioid drugs seem to have a better analgesic effect on women, especially when taken for a long time. Men may need a higher dose to improve the analgesic effect, and thus are more likely to lead to OUD (Romanescu et al., 2022).

Among different countries and regions, the burden of OUD varied significantly. Generally speaking, relatively higher burden of OUD was more frequently seen in high SDI regions, especially in high-income North America. Factors like insufficient regulation of prescription opioid drugs, excessive prescribing of potent opioid drugs, and an open supply chain for illegal opioid drug products may be more common in the developed countries (Fischer et al., 2014; Fisc et al., 2021). Tendency analysis suggests that in high SDI region, OUD burden showed a rapid increasing tendency in recent years. Besides, although the burden of OUD is still relatively low in these low and low-middle SDI regions, the continuous growth trend still needs to be taken seriously. OUD occurs in sub-Saharan Africa and South America, resulting in a significantly rising mortality rate. Evidence-based policies and health system resources are needed to promote OUD prevention and management and reduce the spread of infectious diseases (Kurth et al., 2018).

The present study for the first time used ARIMA models to illustrate global OUD burden trend from 1990 to 2030. The results indicate growth of the global incidence and DALYs of OUD in the next 9 years. In the analysis of the 5 SDI regions, the high SDI and low SDI region also demonstrated increasing trend for ASIR and ASDR. The management of the OUD burden need more attention in the future. In high-SDI regions, the rising trend may be partly attributed to long-standing prescribing practices, aggressive pharmaceutical marketing, and insufficient integration of addiction surveillance systems into healthcare policy (Zhang et al., 2024; Wang et al., 2025). As many low-income countries expand access to opioids for pain management under WHO’s palliative care initiatives, the absence of parallel harm-reduction and surveillance frameworks may inadvertently raise misuse risks (Effatpanah et al., 2025; Coussens et al., 2019). To address these disparities, future global strategies should emphasize context-specific regulatory reforms—tightening pharmacovigilance and diversifying treatment access in high-SDI regions, while building integrated governance, training, and monitoring infrastructure in low-SDI areas.

This study uses a comprehensive, standardized, and globally comparable dataset derived from the GBD study, enabling consistent assessment of OUD burden across diverse populations and over time. By incorporating key epidemiological measures including incidence, prevalence, DALYs, and mortality, our analysis provides a multidimensional understanding of the disease burden from both morbidity and mortality perspectives. However, certain limitations must be acknowledged. First, OUD is defined based on the DSM-IV or ICD-10 diagnostic criteria due to data structure and temporal consistency considerations. However, the introduction of the DSM-5-TR since 2013 eliminates the previous categorization of substance-related addictions into abuse and dependence (Hasin et al., 2013). Consequently, while this approach ensures long-term comparability across the 1990–2021 time series, it may limit direct comparability with newer studies that employ DSM-5 criteria. Furthermore, variations in data quality and completeness across regions may affect the accuracy. Some observed increases in OUD burden might partly reflect improved reporting systems or data availability rather than true epidemiological changes. Although uncertainty intervals were considered, the potential influence of data heterogeneity cannot be fully eliminated. Therefore, our findings should be interpreted with caution, and further real-world studies are necessary to validate our results. In the future, it is essential to continuously update existing information and integrate new data sources to achieve more accurate estimates, which can serve as valuable references for healthcare policymakers.

## 5 Conclusion

This study provides a comprehensive evaluation of the global burden and temporal trends of opioid use disorder (OUD) from 1990 to 2021, integrating decomposition, joinpoint, age–period–cohort, and predictive analyses. Although the absolute number of OUD cases, DALYs, and deaths markedly increased worldwide, the corresponding age-standardized rates remained stable or slightly declined, suggesting that demographic expansion rather than worsening epidemiological risk was the primary driver. Decomposition analysis confirmed that population growth accounted for most of the observed increases, while epidemiological and policy-related factors shaped temporal variations. The joinpoint and APC results revealed a notable decline in OUD burden around 2000, likely reflecting the effects of regulatory measures such as Prescription Drug Monitoring Programs and stricter opioid prescribing policies. However, the resurgence after 2010 may be attributed to the shift from prescription to illicit opioids, particularly in high-income regions. Forecasting analyses predict a modest rise in incidence and DALYs but slight declines in prevalence and mortality by 2030. These findings highlight the complex, evolving nature of the opioid epidemic and emphasize the need for sustained, evidence-based interventions that adapt to demographic shifts, evolving drug markets, and policy environments.

## Data Availability

Publicly available datasets were analyzed in this study. This data can be found here: http://ghdx.healthdata.org/gbd-results-tool.
