# Supplementary material for: Trend analysis and future projections of global burden of opioid use disorder (OUD) from 1990 to 2030
Source: Front Pharmacol. 2025 Nov 25;16:1669269. doi: 10.3389/fphar.2025.1669269 (PMC12685792; doi:10.3389/fphar.2025.1669269)
Supplement: Supplementary file 1 [file Supplementaryfile1.docx]

**Supplementary Table 1. All-age cases, age-standardized rates and EAPC (Estimated Annual Percentage Change) of prevalence for OUD**

| **Location** | **1990** | | **2021** | |  |
| --- | --- | --- | --- | --- | --- |
|  | **Absolute numbers** | **Age-standardized rate (per 100.000)** | **Absolute numbers** | **Age-standardized rate (per 100.000)** | **EAPC** |
| Global | 8120813.5 (6801332.8, 9596422.2) | 154.6 (131.1, 181.3) | 16164875.9 (14133119.8, 18431509.7) | 198.49 (173.42, 227.22) | 0.5 (0.32, 0.69) |
| High SDI | 1943217.7 (1676802.4, 2237423.6) | 202.3 (174.2, 233) | 8316982.4 (7416372.9, 9351503.5) | 761.65 (674.95, 864.14) | 4.5 (4.09, 4.9) |
| High-middle SDI | 2371642.1 (2007552.7, 2789292.2) | 207.2 (176.1, 242.3) | 2208011.1 (1910876.3, 2541927.2) | 159.87 (135.83, 186.5) | -1.43 (-1.84, -1.02) |
| Middle SDI | 2510697.6 (2074323.2, 2991292.8) | 148.7 (125.8, 174.1) | 2832681 (2376556.6, 3335824.1) | 108.64 (90.53, 128.61) | -1.48 (-1.66, -1.3) |
| Low-middle SDI | 983641.6 (771817.5, 1215419.4) | 95.9 (77.8, 116.1) | 2014727.5 (1651316.2, 2416976.9) | 102.41 (85.01, 121.39) | 0.09 (0, 0.18) |
| Low SDI | 307166.9 (240869.4, 380773.3) | 76.8 (61.9, 92.9) | 785959.8 (630138.2, 959125.4) | 80.76 (66.91, 96.27) | 0.1 (0.05, 0.15) |
| Andean Latin America | 34735 (26315.7, 44687.6) | 97.4 (75.4, 121.4) | 70236.8 (54745.3, 86630.9) | 99.23 (77.66, 122.43) | 0.13 (0.04, 0.22) |
| Australasia | 56323.1 (51066.2, 61704.1) | 258 (234.2, 282.9) | 89881.7 (82240, 98308.7) | 284.21 (259.19, 311.84) | 0.13 (-0.19, 0.44) |
| Caribbean | 38655.8 (30318.7, 47884.7) | 108.8 (87.2, 132.3) | 43793.4 (35073.6, 53682.1) | 88.28 (70.52, 108.35) | -0.89 (-1.01, -0.77) |
| Central Asia | 141487.4 (112834.4, 171149.4) | 208.6 (170.1, 248.8) | 213900.7 (186052.1, 247374.6) | 213.65 (185.05, 247.5) | -0.02 (-0.21, 0.17) |
| Central Europe | 94315 (77154.5, 112569.1) | 73.2 (59.5, 88) | 102510.6 (89595, 116807.6) | 89.23 (77.04, 104.1) | 0.6 (0.5, 0.69) |
| Central Latin America | 144016.7 (110256, 180784.5) | 93.4 (73.3, 115.2) | 235672.2 (186457.3, 288977.9) | 87.65 (69.37, 107.42) | -0.21 (-0.31, -0.1) |
| Central Sub-Saharan Africa | 28040.9 (21839.7, 34981.5) | 63.8 (51.1, 78) | 81131.6 (64601.4, 100052.6) | 69.81 (56.86, 83.82) | 0.39 (0.35, 0.43) |
| East Asia | 2438352.1 (2053381, 2881100.2) | 189.2 (161.6, 220) | 1533265.1 (1278054.8, 1804685.4) | 94.72 (77.62, 112.73) | -3.23 (-3.62, -2.85) |
| Eastern Europe | 945656.7 (810645.3, 1103065.9) | 395.7 (340.5, 465.1) | 890549.1 (787614.1, 1008612.3) | 431.53 (379.31, 493.25) | -0.36 (-1.05, 0.33) |
| Eastern Sub-Saharan Africa | 86687.1 (67283.5, 108378.2) | 60.2 (48.4, 72.9) | 214835.7 (171817.1, 262361) | 60.32 (49.34, 71.65) | -0.07 (-0.11, -0.02) |
| High-income Asia Pacific | 177789.1 (139350.9, 216706.1) | 94.8 (74, 116.1) | 162536.2 (132441.3, 194090.5) | 90.22 (71.08, 109.56) | -0.1 (-0.22, 0.02) |
| High-income North America | 1016907 (865277, 1183767.2) | 327.4 (279.2, 381.1) | 6894161.3 (6086133.8, 7821275) | 1890.26 (1659.84, 2156.24) | 6.35 (5.82, 6.88) |
| North Africa and Middle East | 619500.4 (493212.5, 765573) | 198.3 (162, 240.4) | 1456242.5 (1241717.6, 1694858.7) | 222.34 (190.3, 258.56) | 0.44 (0.23, 0.64) |
| Oceania | 3908.7 (3110.2, 4843.6) | 67 (54.2, 80.8) | 9177.5 (7461.9, 11119.9) | 68.86 (56.55, 82.59) | 0.09 (0.07, 0.11) |
| South Asia | 909627.6 (715760.3, 1128915.9) | 93.5 (76.1, 112.7) | 2068682.5 (1684430.7, 2497346.2) | 105.86 (87.16, 126.68) | 0.14 (-0.1, 0.37) |
| Southeast Asia | 230485.6 (186013.6, 280716.6) | 52.6 (43, 62.4) | 405625.6 (339421.2, 477195.9) | 54.3 (45.42, 63.78) | -0.03 (-0.13, 0.07) |
| Southern Latin America | 58848.8 (45275.1, 74008.9) | 120.3 (93, 150.9) | 80166.1 (63224.8, 98118.5) | 110.9 (86.57, 136.22) | -0.3 (-0.38, -0.22) |
| Southern Sub-Saharan Africa | 94461.7 (77621.8, 112190.1) | 202.1 (170.5, 236.6) | 110212.9 (92590.1, 129710.9) | 134.96 (114.3, 157.31) | -1.7 (-2.1, -1.29) |
| Tropical Latin America | 151744 (116449.6, 192061.6) | 99.9 (77.9, 123.5) | 225236 (177263.8, 278465.7) | 90.82 (71.67, 113.42) | -0.27 (-0.4, -0.13) |
| Western Europe | 751195.2 (670343.1, 844808.1) | 183.7 (163.2, 207.1) | 1023831.5 (932726.7, 1126292.3) | 237.54 (213.94, 263.02) | 0.42 (0.12, 0.71) |
| Western Sub-Saharan Africa | 98075.7 (74943, 123314.7) | 62.8 (49.5, 76.9) | 253226.9 (198683.5, 317066.5) | 61.75 (49.74, 74.58) | -0.03 (-0.08, 0.02) |

**Supplementary Table 2. All-age cases, age-standardized rates and EAPC (Estimated Annual Percentage Change) of DALYs** **(Disability-Adjusted Life Years) for OUD**

| **Location** | **1990** | | **2021** | | |
| --- | --- | --- | --- | --- | --- |
|  | **Absolute numbers** | **Age-standardized rate (per 100.000)** | **Absolute numbers** | **Age-standardized rate (per 100.000)** | **EAPCs** |
| Global | 5415249.2 (4242000.6, 6437811.7) | 103.69 (81.83, 122.75) | 11218518.6 (9188657.5, 13159551.4) | 137.15 (112.29, 161.39) | 0.5 (0.3, 0.71) |
| High SDI | 1256300.5 (1007041, 1499490.5) | 130.85 (104.81, 156.09) | 6548594.7 (5421925.8, 7567176.6) | 587.41 (484.84, 680.69) | 4.98 (4.72, 5.24) |
| High-middle SDI | 1560159.8 (1218569.3, 1864510.5) | 136.71 (107.3, 162.8) | 1377430.1 (1108116.5, 1649451.9) | 98.7 (78.06, 119.48) | -1.75 (-2.21, -1.29) |
| Middle SDI | 1872069.8 (1451059.4, 2215513) | 112.76 (88.48, 132.6) | 1667041.8 (1293206.1, 2026943.3) | 63.6 (49.05, 77.63) | -2.5 (-2.76, -2.24) |
| Low-middle SDI | 545169.2 (401808.9, 688249.9) | 54.19 (40.34, 67.01) | 1140801.6 (861828.9, 1406316.2) | 58.69 (44.52, 71.94) | 0.11 (0.01, 0.21) |
| Low SDI | 178840.5 (134815.5, 226787.2) | 46.16 (35.28, 57.74) | 480610.3 (364315.2, 591723.1) | 50.81 (39.15, 61.76) | 0.21 (0.16, 0.27) |
| Andean Latin America | 15492.9 (10234.5, 21241.2) | 43.69 (29.09, 59.24) | 32556.1 (22899.6, 43838.9) | 46.21 (32.59, 61.99) | 0.28 (0.18, 0.39) |
| Australasia | 42362.4 (35245.5, 49108.5) | 194.37 (161.72, 225.02) | 65324.7 (53569.7, 76293.6) | 205.48 (168.06, 240.26) | -0.58 (-1.02, -0.15) |
| Caribbean | 17183.9 (11460.5, 23286.3) | 48.49 (32.7, 64.37) | 20232 (14015.1, 26534.7) | 40.77 (28.15, 53.52) | -1.2 (-1.5, -0.89) |
| Central Asia | 70350 (50798.5, 90742.3) | 104.53 (75.93, 132.93) | 117883.4 (89535.5, 144813) | 117.72 (89.49, 144.38) | 0.35 (-0.08, 0.78) |
| Central Europe | 67672.2 (53454.2, 80857) | 52.45 (41.21, 63.16) | 74952.8 (62010.2, 87477.3) | 64.41 (53.16, 75.8) | 0.49 (0.4, 0.58) |
| Central Latin America | 66015.7 (43818.2, 88785.6) | 43.16 (29.44, 56.57) | 108440.2 (75531.5, 142294.8) | 40.33 (28.12, 52.9) | -0.25 (-0.33, -0.17) |
| Central Sub-Saharan Africa | 17085.4 (12480.6, 22413.6) | 39.45 (28.8, 51.84) | 52140.5 (37993.8, 67999.3) | 45.14 (32.46, 58.02) | 0.52 (0.44, 0.61) |
| East Asia | 1959218.2 (1530331.9, 2320473.6) | 153.51 (120.43, 181.16) | 887517.7 (681703.7, 1086677.9) | 54.37 (40.97, 67.47) | -4.63 (-5.11, -4.15) |
| Eastern Europe | 600805.1 (472699.2, 720761.1) | 250.75 (195.86, 302.22) | 657684.5 (555403.2, 766235.6) | 311.15 (259.01, 365.83) | -0.01 (-0.88, 0.88) |
| Eastern Sub-Saharan Africa | 61833.5 (46368, 79614.1) | 46.11 (34.98, 59.66) | 177705.8 (136840.7, 219976.6) | 51.62 (40.04, 62.98) | 0.29 (0.27, 0.31) |
| High-income Asia Pacific | 80966.7 (54616.4, 106226.5) | 43.18 (29.21, 57.21) | 78890.4 (55977.9, 100982.9) | 43.74 (30.9, 57.08) | -0.01 (-0.21, 0.19) |
| High-income North America | 648336.3 (515178.2, 778216) | 207.65 (165.2, 248.95) | 5570171.4 (4605348.5, 6442015.4) | 1502.44 (1235.96, 1740.1) | 7.06 (6.8, 7.32) |
| North Africa and Middle East | 360787.2 (268099, 461099.1) | 118.35 (89.71, 146.66) | 842161.3 (650327.9, 1031713.7) | 128.78 (99.59, 157.46) | 0.34 (0.13, 0.55) |
| Oceania | 2168.9 (1572.2, 2830.5) | 36.62 (26.83, 47.44) | 4603.3 (3268.2, 6103.5) | 34.29 (24.39, 44.64) | -0.27 (-0.31, -0.22) |
| South Asia | 531401.5 (395554.5, 661800.4) | 55.72 (42.26, 68.1) | 1190532.1 (891410.1, 1478434.7) | 61.71 (46.68, 75.92) | 0.03 (-0.18, 0.24) |
| Southeast Asia | 134442.7 (100396.9, 170086) | 30.74 (23.12, 38.19) | 244550.8 (187103.6, 300794.1) | 32.9 (25.23, 40.51) | 0.09 (-0.01, 0.19) |
| Southern Latin America | 25409 (16199.9, 34156.9) | 51.92 (33.12, 69.72) | 37408.8 (26191.5, 48810.6) | 51.7 (36.07, 67.75) | 0.02 (-0.07, 0.11) |
| Southern Sub-Saharan Africa | 56152.7 (42619.3, 69332.9) | 121.87 (93.86, 147.22) | 71748.9 (57387.9, 85223.3) | 88.95 (71.72, 105.11) | -1.32 (-1.72, -0.92) |
| Tropical Latin America | 62921.4 (40533.6, 86420.9) | 41.29 (27.16, 55.97) | 95394.6 (63997.4, 126549.2) | 38.51 (25.72, 51.57) | -0.15 (-0.27, -0.04) |
| Western Europe | 551604.5 (460041.8, 645176.2) | 136.19 (113.6, 159.53) | 778891.9 (657867.1, 897778.7) | 178.12 (149.99, 207.3) | 0.25 (-0.01, 0.52) |
| Western Sub-Saharan Africa | 43038.8 (28939, 58124.7) | 27.72 (18.96, 36.24) | 109727.5 (72681.3, 147987.8) | 26.84 (18.22, 35.3) | -0.03 (-0.07, 0.02) |

**Supplementary Table 3.** **All-age cases, age-standardized rates and EAPC (Estimated Annual Percentage Change) of mortality for OUD**

| **Location** | **1990** | | **2021** | |  |
| --- | --- | --- | --- | --- | --- |
|  | **Absolute numbers** | **Age-standardized rate (per 100.000)** | **Absolute numbers** | **Age-standardized rate (per 100.000)** | **EAPCs** |
| Global | 41566.5 (36922.9, 45060.1) | 0.86 (0.76, 0.93) | 99555.5 (92947.9, 108049.6) | 1.19 (1.12, 1.29) | 0.86 (0.68, 1.04) |
| High SDI | 8843.3 (8525.2, 9156.1) | 0.91 (0.87, 0.94) | 67688.9 (61163.6, 75435.1) | 5.47 (4.97, 6.06) | 5.94 (5.83, 6.05) |
| High-middle SDI | 11743.4 (10649.4, 12733.2) | 1.07 (0.97, 1.16) | 10285.2 (9480.1, 10983.6) | 0.67 (0.62, 0.71) | -0.94 (-1.45, -0.42) |
| Middle SDI | 16770.4 (13603.3, 19316.6) | 1.16 (0.94, 1.32) | 11215.6 (9703.1, 12660.9) | 0.42 (0.36, 0.47) | -3.33 (-3.76, -2.9) |
| Low-middle SDI | 3053.8 (2623.9, 3415.1) | 0.38 (0.33, 0.43) | 7098.6 (6031.2, 8162) | 0.42 (0.36, 0.48) | 0.26 (0.16, 0.36) |
| Low SDI | 1138.4 (901.5, 1417.7) | 0.38 (0.3, 0.47) | 3236.3 (2472.9, 3940.8) | 0.43 (0.34, 0.51) | 0.33 (0.26, 0.39) |
| Andean Latin America | 21 (16.9, 26.4) | 0.08 (0.06, 0.1) | 77.3 (61.2, 101) | 0.12 (0.09, 0.16) | 1.25 (0.99, 1.51) |
| Australasia | 331.4 (307.5, 357.8) | 1.52 (1.41, 1.64) | 582.3 (507.5, 669.1) | 1.74 (1.51, 1.99) | 0.8 (0.05, 1.56) |
| Caribbean | 22.2 (19.7, 24.8) | 0.07 (0.06, 0.08) | 46.4 (36.7, 56.2) | 0.09 (0.07, 0.11) | 0.96 (-0.43, 2.37) |
| Central Asia | 223.8 (190.7, 263.4) | 0.38 (0.32, 0.45) | 603.4 (496.9, 709.8) | 0.63 (0.52, 0.74) | 1.8 (1.19, 2.42) |
| Central Europe | 598.1 (539.1, 664.1) | 0.45 (0.4, 0.5) | 778.6 (715.1, 845.8) | 0.56 (0.52, 0.61) | 0.88 (0.64, 1.12) |
| Central Latin America | 124.9 (117.4, 132.6) | 0.1 (0.1, 0.11) | 238.7 (206, 277.2) | 0.09 (0.08, 0.1) | -0.83 (-1, -0.66) |
| Central Sub-Saharan Africa | 110.8 (65, 165.3) | 0.3 (0.17, 0.44) | 360.2 (203.3, 548.1) | 0.35 (0.2, 0.53) | 0.36 (0.22, 0.5) |
| East Asia | 19318.1 (15372.1, 22653.3) | 1.68 (1.35, 1.95) | 6019.5 (4808.1, 7354.4) | 0.33 (0.26, 0.4) | -5.34 (-6.03, -4.65) |
| Eastern Europe | 4451.4 (4119.3, 4822.9) | 1.78 (1.64, 1.93) | 6016.2 (5461.8, 6599.4) | 2.55 (2.33, 2.81) | 1.66 (0.9, 2.42) |
| Eastern Sub-Saharan Africa | 550.1 (379.5, 770.5) | 0.56 (0.38, 0.77) | 1785.4 (1210.6, 2304.9) | 0.62 (0.43, 0.79) | 0.37 (0.3, 0.44) |
| High-income Asia Pacific | 140.3 (131.9, 149.2) | 0.07 (0.07, 0.08) | 293.2 (270.1, 314.2) | 0.12 (0.11, 0.13) | 2.08 (1.43, 2.73) |
| High-income North America | 4549.7 (4271.2, 4840.5) | 1.43 (1.34, 1.52) | 58205.5 (51549, 65872.2) | 14.5 (12.92, 16.3) | 7.66 (7.48, 7.84) |
| North Africa and Middle East | 2021.2 (1652.2, 2405.3) | 0.8 (0.65, 0.95) | 4903.4 (4140.3, 5667.2) | 0.81 (0.68, 0.93) | 0.36 (0.21, 0.5) |
| Oceania | 9.8 (6.2, 13.4) | 0.19 (0.12, 0.26) | 14.7 (10.4, 20.1) | 0.13 (0.09, 0.17) | -1.5 (-1.68, -1.32) |
| South Asia | 3483.6 (3011.1, 3937.7) | 0.46 (0.4, 0.53) | 8064.4 (6706.2, 9346.3) | 0.48 (0.4, 0.55) | 0.04 (-0.11, 0.19) |
| Southeast Asia | 759.2 (648.9, 909.9) | 0.21 (0.19, 0.25) | 1684.7 (1371.9, 2077.1) | 0.25 (0.2, 0.3) | 0.45 (0.38, 0.51) |
| Southern Latin America | 22.8 (20.7, 25.1) | 0.05 (0.04, 0.05) | 107.2 (94.9, 123.2) | 0.14 (0.12, 0.16) | 3.39 (2.73, 4.05) |
| Southern Sub-Saharan Africa | 352.3 (299.1, 395.6) | 0.92 (0.77, 1.04) | 618.9 (548.6, 703.7) | 0.88 (0.78, 0.99) | 0.03 (-0.23, 0.29) |
| Tropical Latin America | 11.5 (10.7, 12.3) | 0.01 (0.01, 0.01) | 86.8 (78.8, 95) | 0.03 (0.03, 0.04) | 3.69 (3.1, 4.29) |
| Western Europe | 4393.1 (4256.7, 4528) | 1.06 (1.03, 1.1) | 8949.9 (8426.1, 9403.1) | 1.67 (1.59, 1.74) | 2.82 (2.19, 3.46) |
| Western Sub-Saharan Africa | 71.2 (50.8, 91.6) | 0.07 (0.05, 0.09) | 118.8 (79.3, 153.1) | 0.05 (0.04, 0.07) | -1.49 (-1.93, -1.04) |

**Supplementary Table 4. Joinpoint regression analysis: trends in age-standardized incidence, prevalence, mortality rates (per 100,000 persons) among sexes, males, and females for OUD, 1990–2021.**

| **Gender** | **ASIR** | | | **ASPR** | | | **ASDR** | | | **ASMR** | | |
| --- | --- | --- | --- | --- | --- | --- | --- | --- | --- | --- | --- | --- |
|  | **Period** | **APC（95%CI）** | **AAPC（95%CI）** | **Period** | **APC（95%CI）** | **AAPC（95%CI）** | **Period** | **APC（95%CI）** | **AAPC（95%CI）** | **Period** | **APC（95%CI）** | **AAPC（95%CI）** |
| Both | 1990-2000 | 0.93 (0.83 - 1.03) | 0.12 (-0.04 - 0.29) | 1990-2000 | 0.94 (0.87 - 1.01) | 0.80 (0.71 - 0.90) | 1990-1994 | 2.36 (1.77 - 2.96) | 0.91 (0.68 - 1.15) | 1980-1987 | 0.70 (-0.42 - 1.84) | 1.17 (0.88 - 1.47) |
|  | 2000-2005 | -0.89 (-1.29 - -0.48) |  | 2000-2005 | -0.35 (-0.61 - -0.09) |  | 1994-2006 | -0.02 (-0.14 - 0.11) |  | 1987-1994 | 3.87 (2.69 - 5.07) |  |
|  | 2005-2010 | -2.26 (-2.65 - -1.87) |  | 2005-2010 | -1.25 (-1.51 - -1.00) |  | 2006-2010 | -1.40 (-2.27 - -0.52) |  | 1994-2003 | -1.14 (-1.54 - -0.74) |  |
|  | 2010-2015 | 0.73 (0.33 - 1.13) |  | 2010-2015 | 1.87 (1.61 - 2.12) |  | 2010-2013 | 0.95 (-0.84 - 2.77) |  | 2003-2006 | 1.58 (-0.59 - 3.81) |  |
|  | 2015-2018 | 2.60 (1.34 - 3.87) |  | 2015-2019 | 3.04 (2.66 - 3.43) |  | 2013-2018 | 3.43 (2.85 - 4.00) |  | 2006-2012 | -1.09 (-1.57 - -0.62) |  |
|  | 2018-2021 | -0.27 (-0.88 - 0.34) |  | 2019-2021 | 1.17 (0.43 - 1.90) |  | 2018-2021 | 1.68 (0.82 - 2.55) |  | 2012-2021 | 3.75 (3.45 - 4.05) |  |
| Female | 1990-2001 | -0.10 (-0.14 - -0.06) | 0.11 (0.03 - 0.19) | 1990-2004 | -0.27 (-0.29 - -0.25) | 0.80 (0.76 - 0.85) | 1990-1994 | 0.29 (-0.33 - 0.92) | 0.96 (0.68 - 1.24) | 1980-1995 | 1.05 (0.80 - 1.30) | 1.48 (1.21 - 1.75) |
|  | 2001-2005 | -0.51 (-0.80 - -0.22) |  | 2004-2010 | -0.92 (-1.03 - -0.81) |  | 1994-1997 | -0.88 (-2.78 - 1.05) |  | 1995-1999 | -1.62 (-3.27 - 0.06) |  |
|  | 2005-2010 | -1.80 (-1.99 - -1.62) |  | 2010-2015 | 2.70 (2.55 - 2.85) |  | 1997-2011 | -0.06 (-0.16 - 0.04) |  | 1999-2005 | 2.06 (1.60 - 2.52) |  |
|  | 2010-2015 | 0.64 (0.45 - 0.82) |  | 2015-2019 | 4.61 (4.38 - 4.83) |  | 2011-2015 | 2.95 (2.04 - 3.87) |  | 2005-2013 | 1.24 (1.06 - 1.42) |  |
|  | 2015-2018 | 3.11 (2.52 - 3.70) |  | 2019-2021 | 1.42 (0.99 - 1.85) |  | 2015-2018 | 4.83 (3.02 - 6.67) |  | 2013-2017 | 4.22 (3.47 - 4.97) |  |
|  | 2018-2021 | 1.08 (0.79 - 1.37) |  |  |  |  | 2018-2021 | 2.04 (1.14 - 2.94) |  | 2017-2021 | 2.03 (1.20 - 2.87) |  |
| Male | 1990-1999 | 1.97 (1.78 - 2.15) | 0.14 (-0.05 - 0.32) | 1990-1993 | 2.91 (2.15 - 3.68) | 0.86 (0.73 - 0.99) | 1990-1994 | 3.53 (2.74 - 4.32) | 0.92 (0.56 - 1.28) | 1980-1996 | 2.81 (2.49 - 3.13) | 0.81 (0.42 - 1.19) |
|  | 1999-2003 | -0.51 (-1.47 - 0.46) |  | 1993-2000 | 1.76 (1.52 - 2.01) |  | 1994-2000 | 0.58 (0.06 - 1.11) |  | 1996-2003 | -2.07 (-2.74 - -1.40) |  |
|  | 2003-2011 | -2.15 (-2.40 - -1.90) |  | 2000-2005 | -0.23 (-0.66 - 0.20) |  | 2000-2003 | -1.29 (-3.71 - 1.19) |  | 2003-2006 | 1.43 (-1.29 - 4.22) |  |
|  | 2011-2019 | 1.38 (1.13 - 1.64) |  | 2005-2010 | -1.57 (-1.99 - -1.15) |  | 2003-2006 | 0.88 (-1.58 - 3.40) |  | 2006-2012 | -2.06 (-2.68 - -1.44) |  |
|  | 2019-2021 | -2.40 (-4.16 - -0.60) |  | 2010-2021 | 1.35 (1.27 - 1.44) |  | 2006-2011 | -1.99 (-2.73 - -1.25) |  | 2012-2017 | 4.44 (3.46 - 5.42) |  |
|  |  |  |  |  |  |  | 2011-2021 | 2.26 (2.07 - 2.44) |  | 2017-2021 | 2.40 (1.09 - 3.73) |  |

**Supplementary Table 5. Age-period cohort analysis of OUD incidence rate in China, 1990-2021**

| Age | Rate | 95% CI | |
| --- | --- | --- | --- |
|  |  | Lower | Upper |
| 17.5 | 63.699 | 60.926 | 66.597 |
| 22.5 | 108.572 | 103.955 | 113.394 |
| 27.5 | 64.768 | 62.015 | 67.643 |
| 32.5 | 37.23 | 35.64 | 38.891 |
| 37.5 | 27.498 | 26.325 | 28.722 |
| 42.5 | 21.882 | 20.949 | 22.856 |
| 47.5 | 20.507 | 19.706 | 21.34 |
| 52.5 | 17.696 | 16.993 | 18.428 |
| 57.5 | 13.447 | 12.873 | 14.047 |
| 62.5 | 10.698 | 10.193 | 11.229 |
| 67.5 | 9.436 | 8.931 | 9.969 |
| 72.5 | 8.614 | 8.077 | 9.186 |
| 77.5 | 8.204 | 7.575 | 8.885 |
| 82.5 | 8.198 | 7.44 | 9.034 |
| 87.5 | 8.71 | 7.675 | 9.884 |
| 92.5 | 9.602 | 7.958 | 11.586 |
| 97.5 | 10.365 | 7.474 | 14.374 |
|  |  | 95% CI | |
| Period | Rate Ratio | Lower | Upper |
| 1994.5 | 1.016 | 0.993 | 1.04 |
| 1999.5 | 1.044 | 1.026 | 1.063 |
| 2004.5 | 1 | 1 | 1 |
| 2009.5 | 0.895 | 0.879 | 0.911 |
| 2014.5 | 0.874 | 0.854 | 0.895 |
| 2019.5 | 0.896 | 0.869 | 0.924 |
|  |  | 95% CI | |
| Cohort | Rate Ratio | Lower | Upper |
| 1897 | 1.393 | 0.445 | 4.358 |
| 1902 | 1.304 | 0.784 | 2.168 |
| 1907 | 1.298 | 0.982 | 1.717 |
| 1912 | 1.35 | 1.127 | 1.618 |
| 1917 | 1.38 | 1.208 | 1.577 |
| 1922 | 1.371 | 1.24 | 1.517 |
| 1927 | 1.311 | 1.209 | 1.421 |
| 1932 | 1.208 | 1.128 | 1.294 |
| 1937 | 1.12 | 1.056 | 1.189 |
| 1942 | 1.043 | 0.989 | 1.099 |
| 1947 | 1 | 1 | 1 |
| 1952 | 0.998 | 0.955 | 1.042 |
| 1957 | 0.95 | 0.911 | 0.992 |
| 1962 | 0.888 | 0.851 | 0.927 |
| 1967 | 0.842 | 0.806 | 0.878 |
| 1972 | 0.818 | 0.784 | 0.854 |
| 1977 | 0.815 | 0.78 | 0.851 |
| 1982 | 0.822 | 0.786 | 0.859 |
| 1987 | 0.809 | 0.774 | 0.846 |
| 1992 | 0.798 | 0.762 | 0.834 |
| 1997 | 0.825 | 0.788 | 0.864 |
| 2002 | 0.873 | 0.83 | 0.919 |

**Supplementary Table 6. Wald Test Statistics Table for the Age-Period-Cohort Model of OUD Incidence**

| **Test Description** | **Incidence** | | |
| --- | --- | --- | --- |
|  | **X2** | **df** | **P-Value** |
| NetDrift = 0 | 67.5771 | 1 | < 0.0001 |
| All Age Deviations = 0 | 14234.4373 | 15 | < 0.0001 |
| All Period Deviations = 0 | 192.169 | 4 | < 0.0001 |
| All Cohort Deviations = 0 | 241.208 | 20 | < 0.0001 |
| All Period RR = 1 | 258.2854 | 5 | < 0.0001 |
| All Cohort RR = 1 | 317.015 | 21 | < 0.0001 |
| All Local Drifts = Net Drift | 224.8868 | 17 | < 0.0001 |

**Supplementary Table 7. Changes in incidence number according to Age-Period-Cohort models from 1990 to 2021**

| **Location** | **Sex** | **Overall difference** | **Change due to Population-level determinants(% contribute to the total change)** | | |
| --- | --- | --- | --- | --- | --- |
|  |  |  | **Aging** | **Population** | **Epidemiological change** |
| Global | Both | 5847397 | -20.81 | 77.26 | 43.55 |
|  | Female | 2670266 | -20.63 | 81.55 | 39.08 |
|  | Male | 3162601 | -20.92 | 73.62 | 47.3 |
| High SDI | Both | 426331.5 | -40.13 | 74.65 | 65.48 |
|  | Female | 185379.1 | -38.99 | 79.13 | 59.86 |
|  | Male | 239740 | -41.74 | 71.43 | 70.31 |
| High-middle SDI | Both | 201592 | -97.98 | 140.09 | 57.89 |
|  | Female | 91377.78 | -94.73 | 152.9 | 41.84 |
|  | Male | 107407.2 | -105.89 | 130.82 | 75.07 |
| Middle SDI | Both | 1555364 | -28.99 | 78.14 | 50.85 |
|  | Female | 715335.9 | -28.9 | 83.47 | 45.42 |
|  | Male | 838409.4 | -28.49 | 73.15 | 55.34 |
| Low-middle SDI | Both | 1907038 | -12.33 | 76.3 | 36.03 |
|  | Female | 882210 | -12.2 | 80.05 | 32.15 |
|  | Male | 1019568 | -12.27 | 72.97 | 39.29 |
| Low SDI | Both | 1459661 | 5.12 | 73.8 | 21.09 |
|  | Female | 681497.2 | 4.92 | 76.06 | 19.01 |
|  | Male | 771369.7 | 5.4 | 71.81 | 22.79 |

**Supplementary Table 8. Changes in prevalence number according to Age-Period-Cohort models from 1990 to 2021**

| **Location** | **Sex** | **Overall difference** | **Change due to Population-level determinants(% contribute to the total change)** | | |
| --- | --- | --- | --- | --- | --- |
|  |  |  | **Aging** | **Population** | **Epidemiological change** |
| Global | Both | 50167357 | 8.17 | 52.49 | 39.34 |
|  | Female | 25135958 | 8.77 | 52.71 | 38.52 |
|  | Male | 24816201 | 7.54 | 51.6 | 40.86 |
| High SDI | Both | 6609237 | 9.36 | 34.46 | 56.18 |
|  | Female | 3286611 | 9.66 | 33.12 | 57.22 |
|  | Male | 3309071 | 8.94 | 35.28 | 55.77 |
| High-middle SDI | Both | 8578818 | -3.23 | 31.71 | 71.51 |
|  | Female | 4263716 | -1.77 | 31.33 | 70.44 |
|  | Male | 4301294 | -4.26 | 31.61 | 72.66 |
| Middle SDI | Both | 15394715 | 15.56 | 47.38 | 37.07 |
|  | Female | 7747639 | 16.38 | 47.82 | 35.8 |
|  | Male | 7570444 | 14.66 | 46.41 | 38.93 |
| Low-middle SDI | Both | 12053487 | 7.47 | 62.55 | 29.98 |
|  | Female | 6049417 | 8.1 | 63.19 | 28.71 |
|  | Male | 5933381 | 6.9 | 61.17 | 31.93 |
| Low SDI | Both | 6884411 | 0.88 | 80.89 | 18.24 |
|  | Female | 3414944 | 1.27 | 81.08 | 17.65 |
|  | Male | 3413687 | 0.43 | 80.25 | 19.31 |

**Supplementary Table 9. Changes in DALYs (Disability-Adjusted Life Years) number according to Age-Period-Cohort models from 1990 to 2021**

| **Location** | **Sex** | **Overall difference** | **Change due to Population-level determinants(% contribute to the total change)** | | |
| --- | --- | --- | --- | --- | --- |
|  |  |  | **Aging** | **Population** | **Epidemiological change** |
| Global | Both | 51379072 | -1.51 | 43.42 | 58.09 |
|  | Female | 20676011 | -0.9 | 42.92 | 57.99 |
|  | Male | 30562665 | -1.66 | 43.72 | 57.94 |
| High SDI | Both | 5691370 | -6.33 | 31.87 | 74.46 |
|  | Female | 2276306 | -4.99 | 29.87 | 75.12 |
|  | Male | 3437845 | -6.92 | 33.61 | 73.31 |
| High-middle SDI | Both | 4946560 | -49.56 | 44.17 | 105.39 |
|  | Female | 2059810 | -44.07 | 40.77 | 103.3 |
|  | Male | 2915296 | -52.19 | 46.8 | 105.4 |
| Middle SDI | Both | 16216820 | 7.9 | 36.67 | 55.43 |
|  | Female | 6553670 | 8.45 | 36.7 | 54.85 |
|  | Male | 9589785 | 7.74 | 36.46 | 55.8 |
| Low-middle SDI | Both | 14892639 | 8.51 | 44.24 | 47.25 |
|  | Female | 5957967 | 8.11 | 44.57 | 47.31 |
|  | Male | 8853599 | 9.09 | 43.83 | 47.07 |
| Low SDI | Both | 7506612 | 6.39 | 55.03 | 38.58 |
|  | Female | 3018610 | 6.04 | 55.08 | 38.88 |
|  | Male | 4416262 | 6.74 | 55.05 | 38.21 |

**Supplementary Table 10. The optimized model and AIC for ARIMA models for OUD**

| **Location** | **Incidence** | | **Prevalence** | | **DALYs** | | **Mortality** | |
| --- | --- | --- | --- | --- | --- | --- | --- | --- |
|  | **Model** | **AIC** | **Model** | **AIC** | **Model** | **AIC** | **Model** | **AIC** |
| Global | (1,1,0) | -3.59 | (1,1,0) | 116.6 | (2,1,1) | 159.42 | (0,1,2) | -70.12 |
| High SDI | (0,2,2) | 123.33 | (0,2,0) | 214.81 | (0,1,0) | 302.74 | (1,1,0) | 93.21 |
| High-middle SDI | (3,2,0) | -26.74 | (0,2,1) | 104.31 | (2,2,3) | 199.07 | (0,1,0) | -9.78 |
| Middle SDI | (2,1,0) | -44.06 | (2,1,0) | 71.41 | (0,1,4) | 219.82 | (0,1,1) | -4.14 |
| Low-middle SDI | (2,0,2) | -108.86 | (2,0,2) | 0.37 | (1,0,3) | 111.07 | (1,0,0) | -181.93 |
| Low SDI | (1,1,2) | -153.99 | (1,1,2) | -44.81 | (1,1,0) | 55.51 | (0,1,0) | -181.76 |

**Supplementary Table 11. The white noise test for ARIMA models for OUD**

| **Location** | **Incidence** | | **Prevalence** | | **DALYs** | | **Mortality** | |
| --- | --- | --- | --- | --- | --- | --- | --- | --- |
|  | **χ2** | **p-value** | **χ2** | **p-value** | **χ2** | **p-value** | **χ2** | **p-value** |
| Global | 0.24955 | 0.6174 | 0.010353 | 0.919 | 0.29339 | 0.5881 | 0.0054104 | 0.9414 |
| High SDI | 0.0029991 | 0.9563 | 0.27518 | 0.5999 | 1.8887 | 0.1693 | 0.78274 | 0.3763 |
| High-middle SDI | 0.0083168 | 0.9273 | 0.04589 | 0.8304 | 0.79902 | 0.3714 | 2.9625 | 0.08522 |
| Middle SDI | 0.085465 | 0.77 | 0.044448 | 0.833 | 0.53153 | 0.466 | 0.051337 | 0.8208 |
| Low-middle SDI | 0.025787 | 0.8724 | 0.00012617 | 0.991 | 0.091098 | 0.7628 | 0.51504 | 0.473 |
| Low SDI | 0.079521 | 0.7779 | 0.11756 | 0.7317 | 0.00019627 | 0.9888 | 0.185 | 0.6671 |

**Supplementary Table 12. The predictive capacity of ARIMA for OUD**

| **Measure** | **Location** | **ME** | **RMSE** | **MAE** | **MPE** | **MAPE** | **MASE** |
| --- | --- | --- | --- | --- | --- | --- | --- |
| ASIR | Global | 0.009422872 | 0.2076511 | 0.1454528 | 0.04830643 | 0.6055546 | 0.4903066 |
|  | High SDI | 0.2377432 | 1.633042 | 0.8097806 | 0.547955 | 1.483486 | 0.4607524 |
|  | High-middle SDI | -0.01167707 | 0.1277676 | 0.09082632 | -0.01419998 | 0.2718286 | 0.1155283 |
|  | Middle SDI | -0.02378003 | 0.1017357 | 0.06678598 | -0.1051473 | 0.3263833 | 0.2907406 |
|  | Low-middle SDI | 0.001250163 | 0.03085646 | 0.02174063 | 0.00667377 | 0.1237209 | 0.1781877 |
|  | Low SDI | 0.003670427 | 0.01535646 | 0.01258178 | 0.02641794 | 0.08921352 | 0.2740778 |
| ASPR | Global | 0.1630778 | 1.436396 | 0.8903008 | 0.1075503 | 0.5040829 | 0.4004718 |
|  | High SDI | -0.174043 | 8.130948 | 3.334485 | 0.1352583 | 0.6191598 | 0.1841037 |
|  | High-middle SDI | -0.1802296 | 1.235276 | 0.8656492 | -0.05616969 | 0.4448665 | 0.1877473 |
|  | Middle SDI | -0.1594084 | 0.6548631 | 0.4348391 | -0.1174801 | 0.3602721 | 0.2686956 |
|  | Low-middle SDI | 0.00724702 | 0.170318 | 0.1175268 | 0.006910207 | 0.1189981 | 0.1766725 |
|  | Low SDI | 0.02119591 | 0.09014727 | 0.07111096 | 0.02718383 | 0.08948634 | 0.2514183 |
| ASDR | Global | 0.4268086 | 2.70964 | 2.016236 | 0.3480311 | 1.748481 | 0.882748 |
|  | High SDI | 0.00362873 | 29.47711 | 17.76685 | -1.569903 | 5.595314 | 0.7627298 |
|  | High-middle SDI | -0.79338 | 4.547762 | 3.217848 | -0.4226391 | 2.407647 | 0.596054 |
|  | Middle SDI | -1.909363 | 6.989106 | 4.358582 | -2.62019 | 5.308225 | 0.9366978 |
|  | Low-middle SDI | 0.06042628 | 1.119508 | 0.8570737 | 0.06624366 | 1.513981 | 0.894316 |
|  | Low SDI | 0.0021252 | 0.5274545 | 0.3942152 | -0.0002943612 | 0.8107242 | 0.8371558 |
| ASMR | Global | 0.006627728 | 0.0682705 | 0.04994835 | 0.4148586 | 5.048214 | 0.8239161 |
|  | High SDI | 0.02829942 | 0.9940949 | 0.5846014 | -9.770073 | 29.39162 | 0.6375574 |
|  | High-middle SDI | -0.01296428 | 0.1969361 | 0.1197275 | -3.557755 | 11.77583 | 0.9690201 |
|  | Middle SDI | -0.01787488 | 0.2079513 | 0.1139519 | -7.949989 | 17.33818 | 0.8436622 |
|  | Low-middle SDI | 0.0004493972 | 0.01278361 | 0.01026094 | 0.0113714 | 2.538231 | 0.9086291 |
|  | Low SDI | 0.0006162274 | 0.01229456 | 0.009557841 | 0.1125061 | 2.371074 | 0.9699512 |

**Supplementary Table 13. The actual and forecast values for OUD**

| **Measure** | **Location** | **Actual (2021)** | | | **Forecast (2030)** | | |
| --- | --- | --- | --- | --- | --- | --- | --- |
|  |  | **Rate** | **95% CI Lower** | **95% CI** Upper | **Rate** | **95% CI Lower** | **95% CI Upper** |
| ASIR | Global | 24.54426 | 20.73915 | 29.47552 | 25.13938 | 21.25698 | 29.02179 |
|  | High SDI | 68.5239 | 57.66505 | 82.32535 | 92.99819 | 67.12002 | 118.87635 |
|  | High-middle SDI | 27.16473 | 23.01557 | 32.63073 | 26.40436 | 14.54677 | 38.26195 |
|  | Middle SDI | 18.81703 | 15.80929 | 22.76383 | 17.71352 | 13.68505 | 21.74199 |
|  | Low-middle SDI | 18.21762 | 15.29222 | 22.17016 | 17.41341 | 16.54019 | 18.28664 |
|  | Low SDI | 14.35181 | 11.92664 | 17.42377 | 14.63990 | 13.95356 | 15.32625 |
| ASPR | Global | 198.4887 | 173.4231 | 227.2182 | 193.1991 | 162.1981 | 224.2001 |
|  | High SDI | 761.6506 | 674.9545 | 864.1355 | 752.1977 | 474.3377 | 1030.0577 |
|  | High-middle SDI | 159.8735 | 135.8343 | 186.5018 | 132.7938 | 65.39631 | 200.1913 |
|  | Middle SDI | 108.6429 | 90.53414 | 128.6121 | 100.5529 | 73.99442 | 127.1114 |
|  | Low-middle SDI | 102.4072 | 85.00736 | 121.3856 | 97.93418 | 93.05236 | 102.8160 |
|  | Low SDI | 80.75554 | 66.90518 | 96.26562 | 83.43792 | 78.82805 | 88.04780 |
| ASDR | Global | 137.1459 | 112.2928 | 161.3851 | 145.8116 | 128.0171 | 163.6060 |
|  | High SDI | 587.4104 | 484.8356 | 680.6863 | 719.9610 | 540.9546 | 898.9675 |
|  | High-middle SDI | 98.69693 | 78.05958 | 119.4773 | 76.34145 | 4.03583 | 148.6471 |
|  | Middle SDI | 63.59549 | 49.05239 | 77.63209 | 63.32791 | 26.02457 | 100.63124 |
|  | Low-middle SDI | 58.69024 | 44.51866 | 71.94097 | 56.64301 | 53.70451 | 59.58152 |
|  | Low SDI | 50.80998 | 39.15261 | 61.75728 | 52.09426 | 49.73037 | 54.45816 |
| ASMR | Global | 1.193758 | 1.115365 | 1.293866 | 1.0327366 | 0.7091401 | 1.356333 |
|  | High SDI | 5.466664 | 4.966623 | 6.056144 | 1.466498 | -2.4534886 | 5.386484 |
|  | High-middle SDI | 0.667857 | 0.615748 | 0.714626 | 0.6521457 | -0.52434562 | 1.828637 |
|  | Middle SDI | 0.419224 | 0.363688 | 0.473039 | 0.8822273 | 0.1406702 | 1.623784 |
|  | Low-middle SDI | 0.417573 | 0.356962 | 0.478259 | 0.4050518 | 0.3753440 | 0.4347596 |
|  | Low SDI | 0.42598 | 0.335272 | 0.512646 | 0.3981098 | 0.3246624 | 0.4715572 |

**Supplementary figure 1** The age-time correlation analysis of OUD incidence

**Supplementary figure 2** The age-time correlation analysis of OUD prevalence

**Supplementary figure 3** The age-time correlation analysis of OUD DALYs

**Supplementary figure 4** The age-time correlation analysis of OUD mortality

**Supplementary figure 5** The sex-time correlation analysis of OUD incidence

**Supplementary figure 6** The sex-time correlation analysis of OUD prevalence

**Supplementary figure 7** The sex-time correlation analysis of OUD DALYs

**Supplementary figure 8** The sex-time correlation analysis of OUD mortality

**Supplementary figure 9** The correlation between SDI and OUD burden in the 21 GBD regions in 2021: (A) age-standardized incidence rate (ASIR); (B) age-standardized prevalence rate (ASPR); (C) age-standardized DALYs rate (ASDR); (D) age-standardized mortality rate (ASMR).

**Supplementary figure 10** The correlation between SDI and OUD burden in the 204 GBD countries and regions in 2021: (A) age-standardized incidence rate (ASIR); (B) age-standardized prevalence rate (ASPR); (C) age-standardized DALYs rate (ASDR); (D) age-standardized mortality rate (ASMR).

**Supplementary figure 12** The age-period-cohort (APC) analysis of OUD incidence

**Supplementary figure 13** Predicted trend of age-standardized prevalence rate (ASPR) from 1990 to 2030 for OUD: (A) Global; (B) High SDI region; (C) High-middle Region; (D) Middle region; (E) Low-middle region; (F) Low SDI region

**Supplementary figure 9** The Joinpoint analysis results of OUD burden: (A) Incidence; (B) Prevalence; (C); DALYs; (D) Mortality
