# Supplementary material for: Trend analysis and future projections of global burden of opioid use disorder (OUD) from 1990 to 2030
Source: Front Pharmacol. 2025 Nov 25;16:1669269. doi: 10.3389/fphar.2025.1669269 (PMC12685792; doi:10.3389/fphar.2025.1669269)
Supplement: Supplementary file 2 [file Supplementaryfile2.docx]

Supplementary figure 1 The age-time correlation analysis of opioid use disorder (OUD) incidence





Supplementary figure 2 The age-time correlation analysis of opioid use disorder (OUD) prevalence





Supplementary figure 3 The age-time correlation analysis of opioid use disorder (OUD) DALYs





Supplementary figure 4 The age-time correlation analysis of opioid use disorder (OUD) mortality





Supplementary figure 5 The sex-time correlation analysis of opioid use disorder (OUD) incidence





Supplementary figure 6 The sex-time correlation analysis of opioid use disorder (OUD) prevalence





Supplementary figure 7 The sex-time correlation analysis of opioid use disorder (OUD) DALYs





Supplementary figure 8 The sex-time correlation analysis of opioid use disorder (OUD) mortality


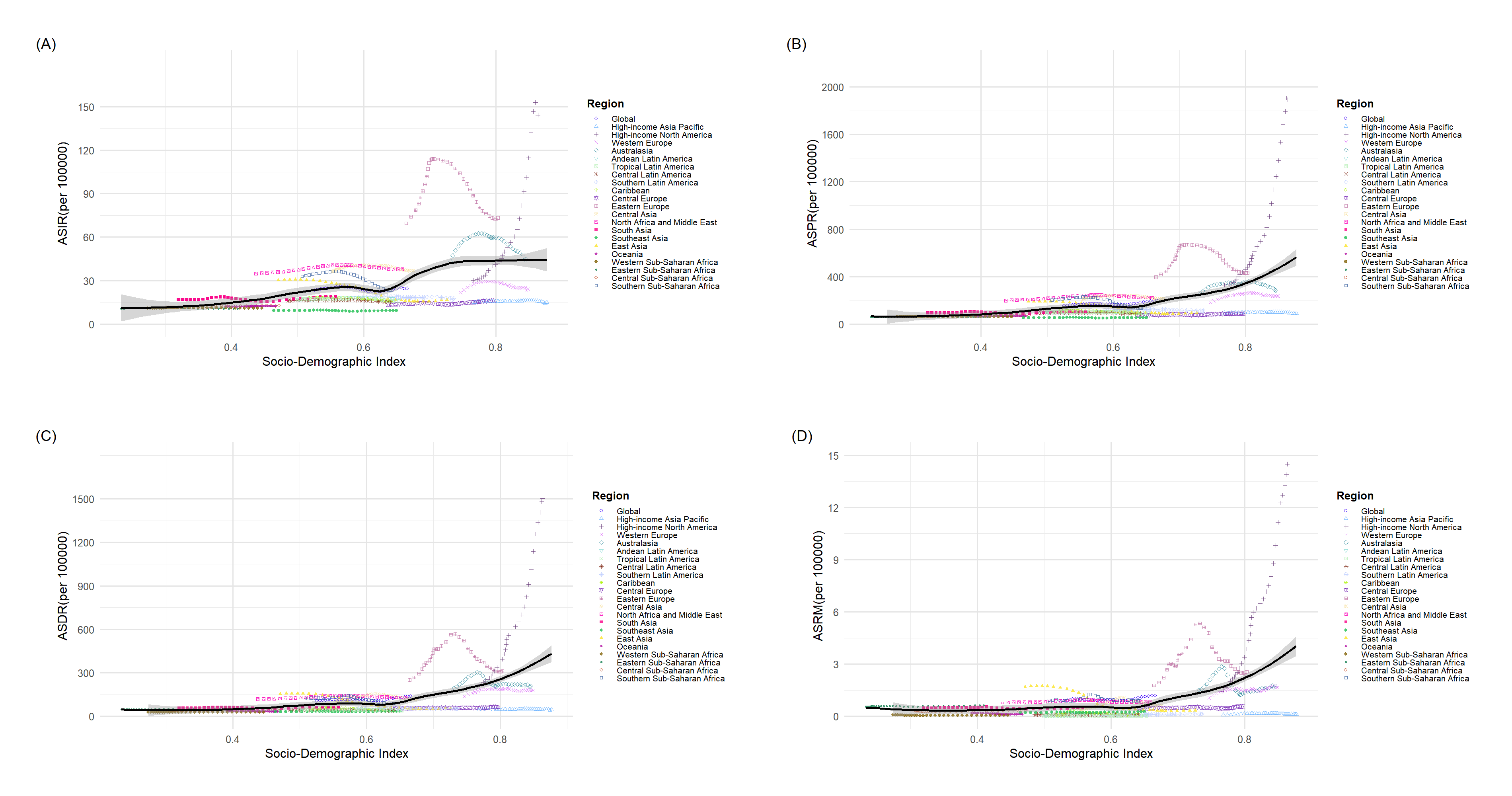


Supplementary figure 9 The correlation between SDI and opioid use disorder (OUD) burden in the 21 GBD regions in 2021: (A) age-standardized incidence rate (ASIR); (B) age-standardized prevalence rate (ASPR); (C) age-standardized DALYs rate (ASDR); (D) age-standardized mortality rate (ASMR).


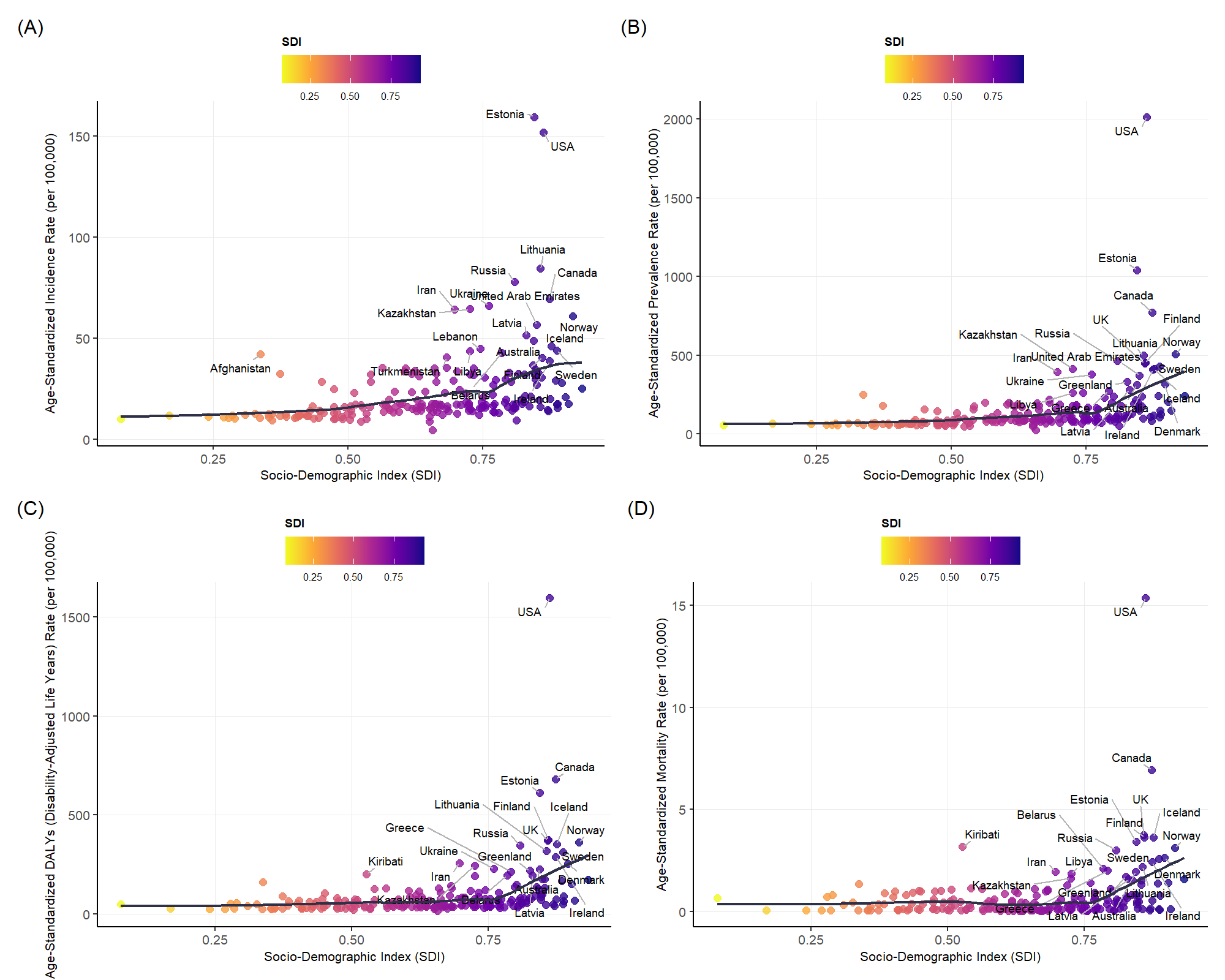


Supplementary figure 10 The correlation between SDI and opioid use disorder (OUD) burden in the 204 GBD countries and regions in 2021: (A) age-standardized incidence rate (ASIR); (B) age-standardized prevalence rate (ASPR); (C) age-standardized DALYs rate (ASDR); (D) age-standardized mortality rate (ASMR).





Supplementary figure 11 Predicted trend of age-standardized prevalence rate (ASPR) from 1990 to 2030 for opioid use disorder (OUD): (A) Global; (B) High SDI region; (C) High-middle Region; (D) Middle region; (E) Low-middle region; (F) Low SDI region


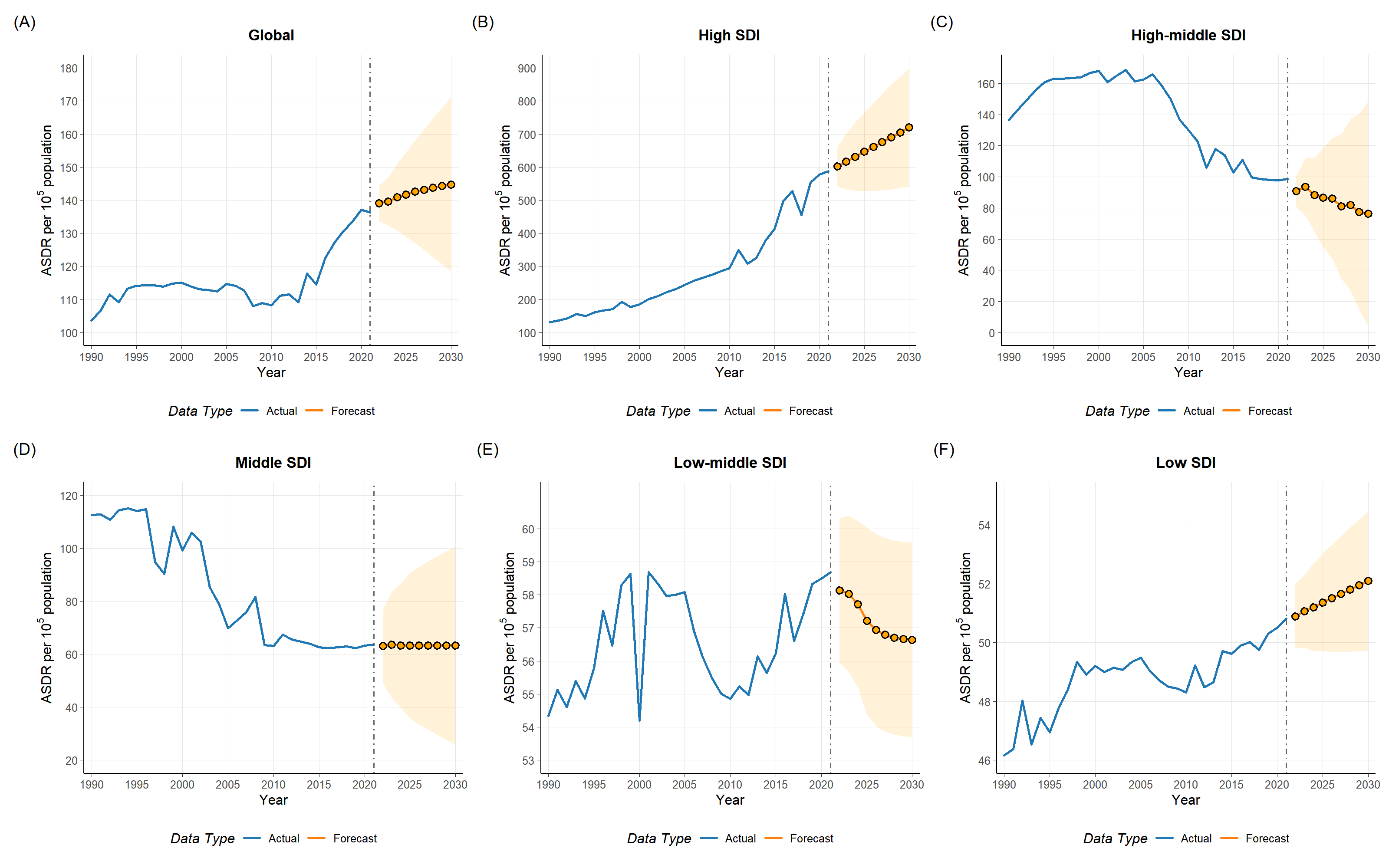


Supplementary figure 12 Predicted trend of age-standardized DALYs rate (ASDR) from 1990 to 2030 for opioid use disorder (OUD): (A) Global; (B) High SDI region; (C) High-middle Region; (D) Middle region; (E) Low-middle region; (F) Low SDI region


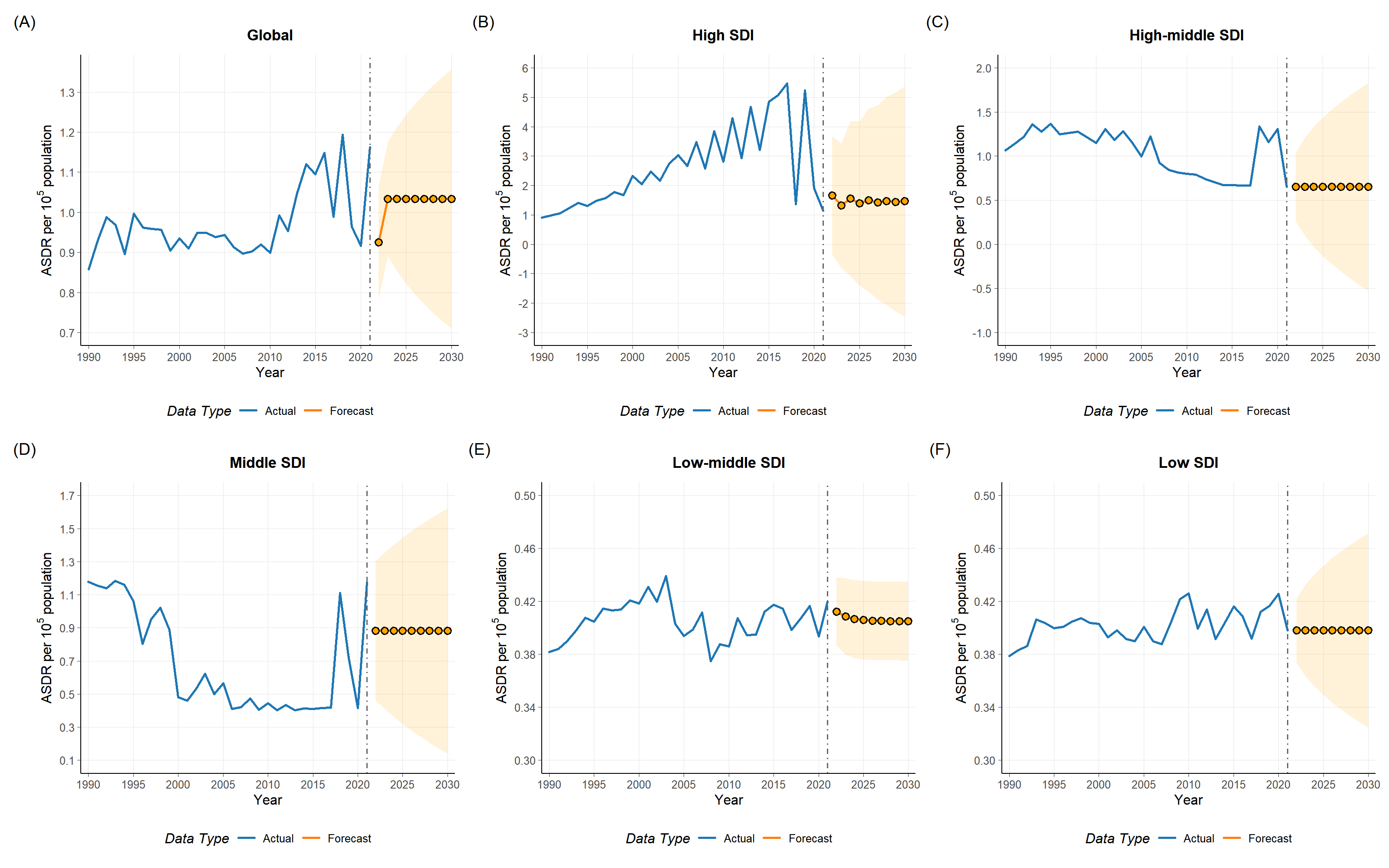


Supplementary figure 13 Predicted trend of age-standardized mortality rate (ASMR) from 1990 to 2030 for opioid use disorder (OUD): (A) Global; (B) High SDI region; (C) High-middle Region; (D) Middle region; (E) Low-middle region; (F) Low SDI region


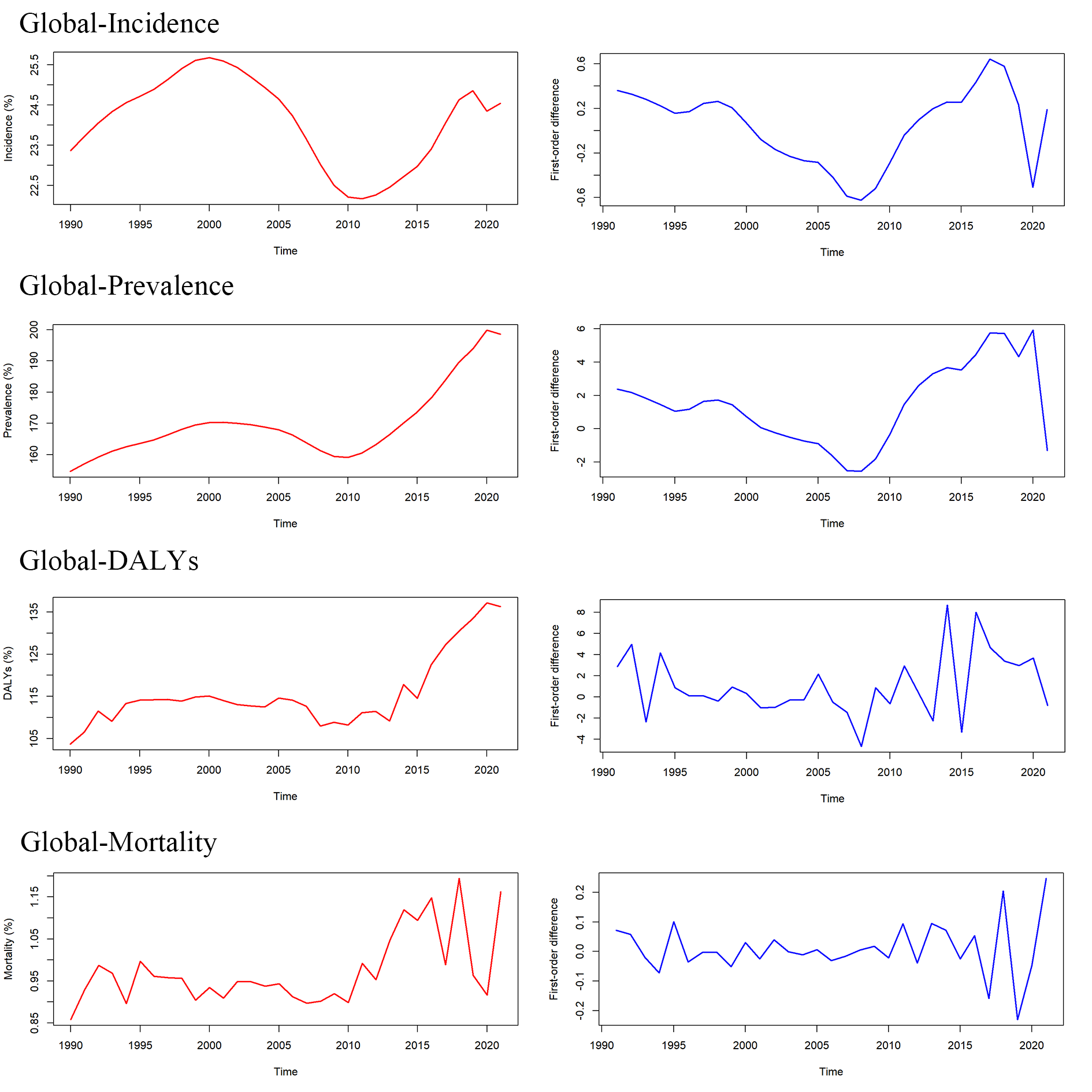


Supplementary figure 14 Timing diagram of global opioid use disorder (OUD) incidence, prevalence,DALYs and mortality (red lines: OUD incidence, prevalence, DALYs and mortality rate; blue lines: OUD incidence, prevalence, DALYs and mortality after differencing)


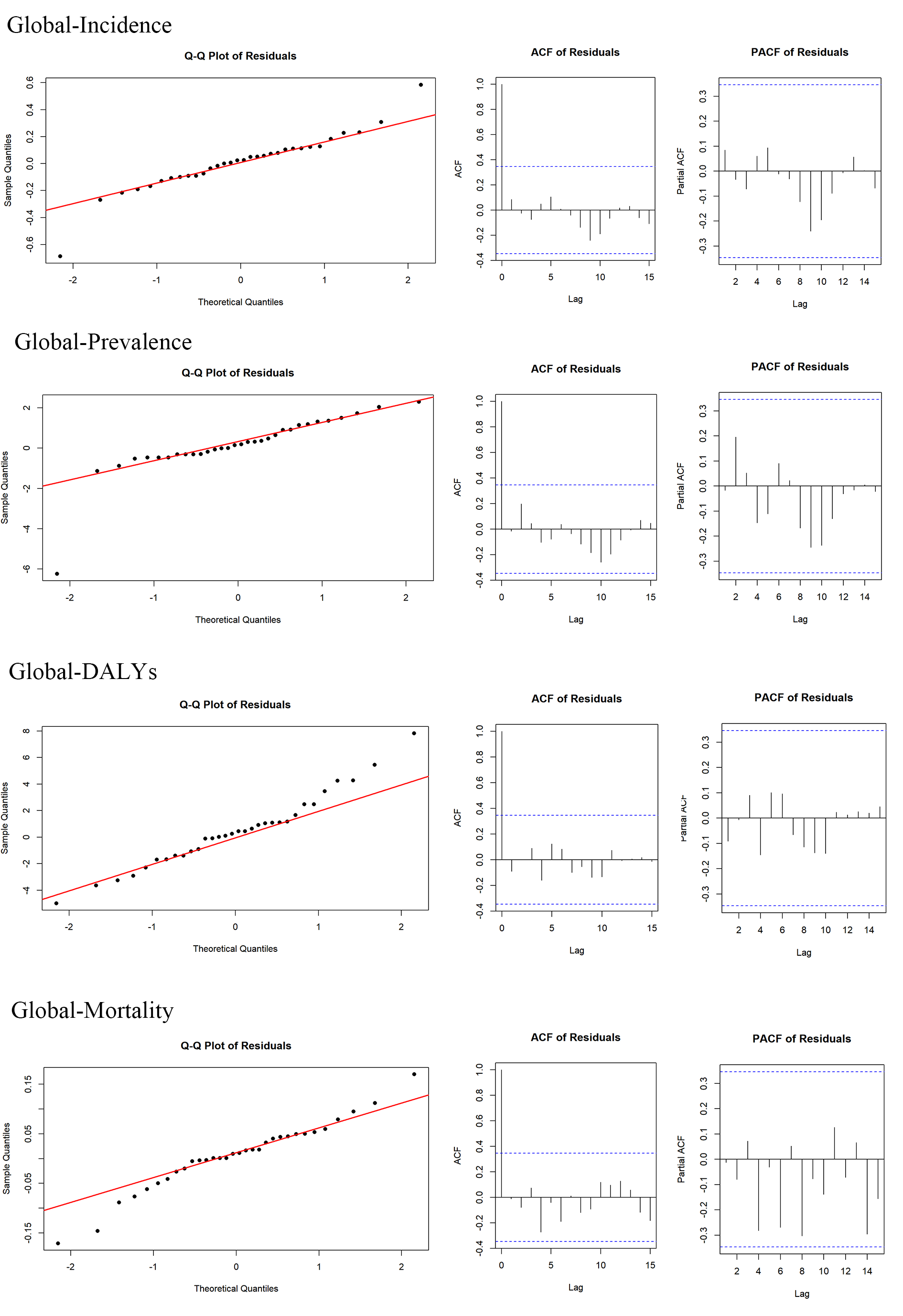


Supplementary figure 15 Residual Q-Q plots, autocorrelation function and partial autocorrelation graphs of the ARIMA models globally


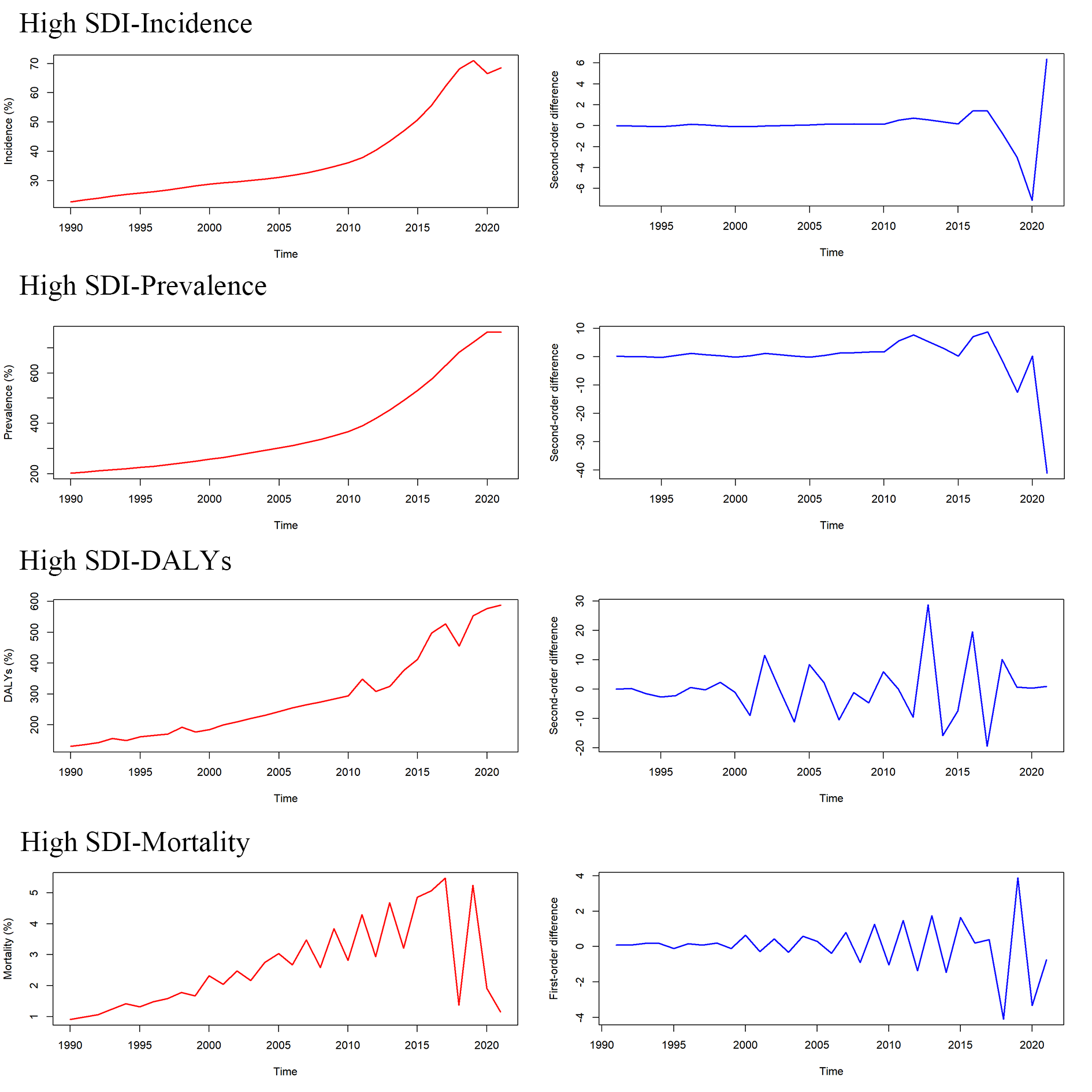


Supplementary figure 16 Timing diagram of opioid use disorder (OUD) incidence in high SDI region, prevalence,DALYs and mortality (red lines: OUD incidence, prevalence, DALYs and mortality rate; blue lines: OUD incidence, prevalence, DALYs and mortality after differencing)


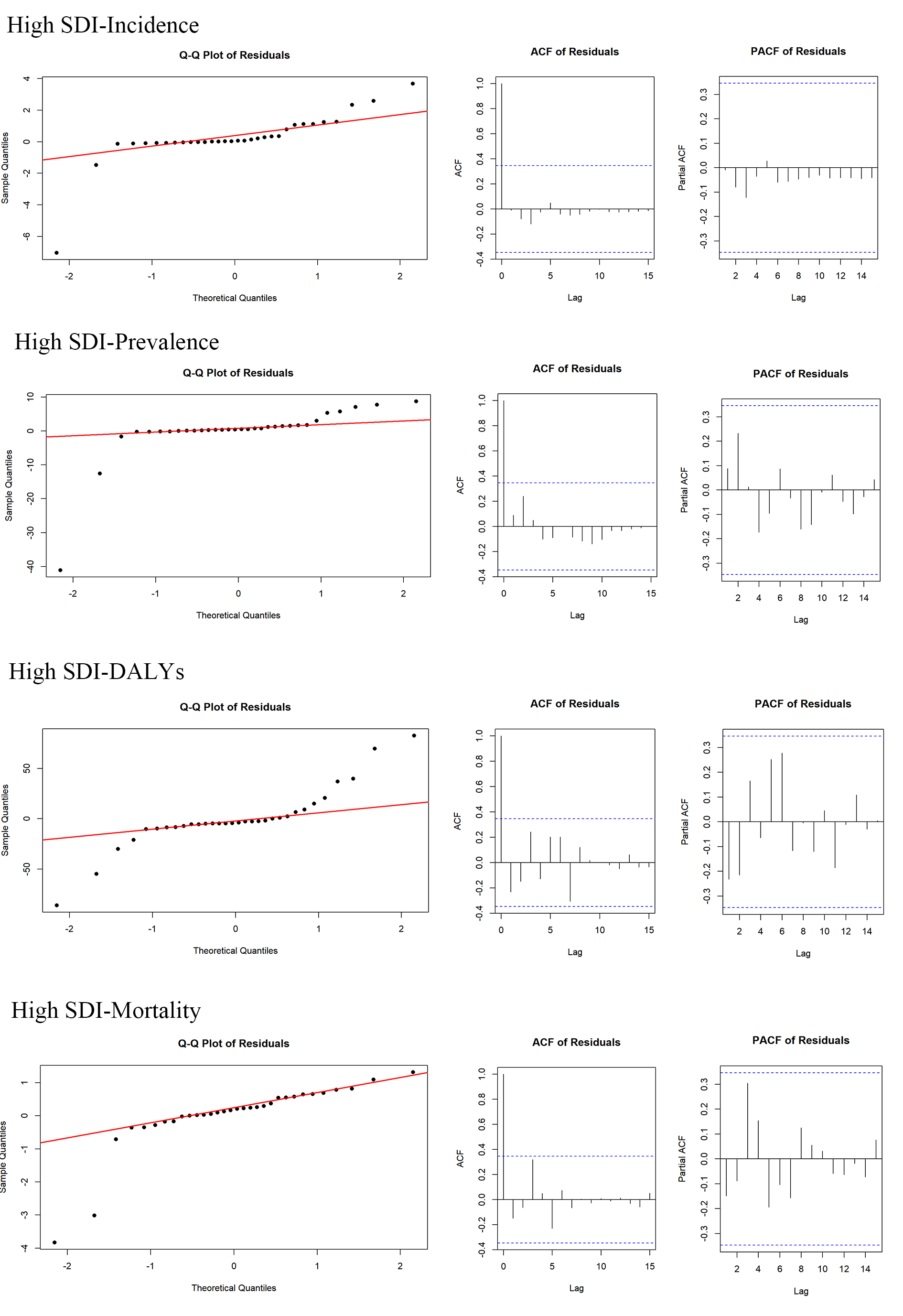


Supplementary figure 17 Residual Q-Q plots, autocorrelation function and partial autocorrelation graphs of the ARIMA models for high SDI region


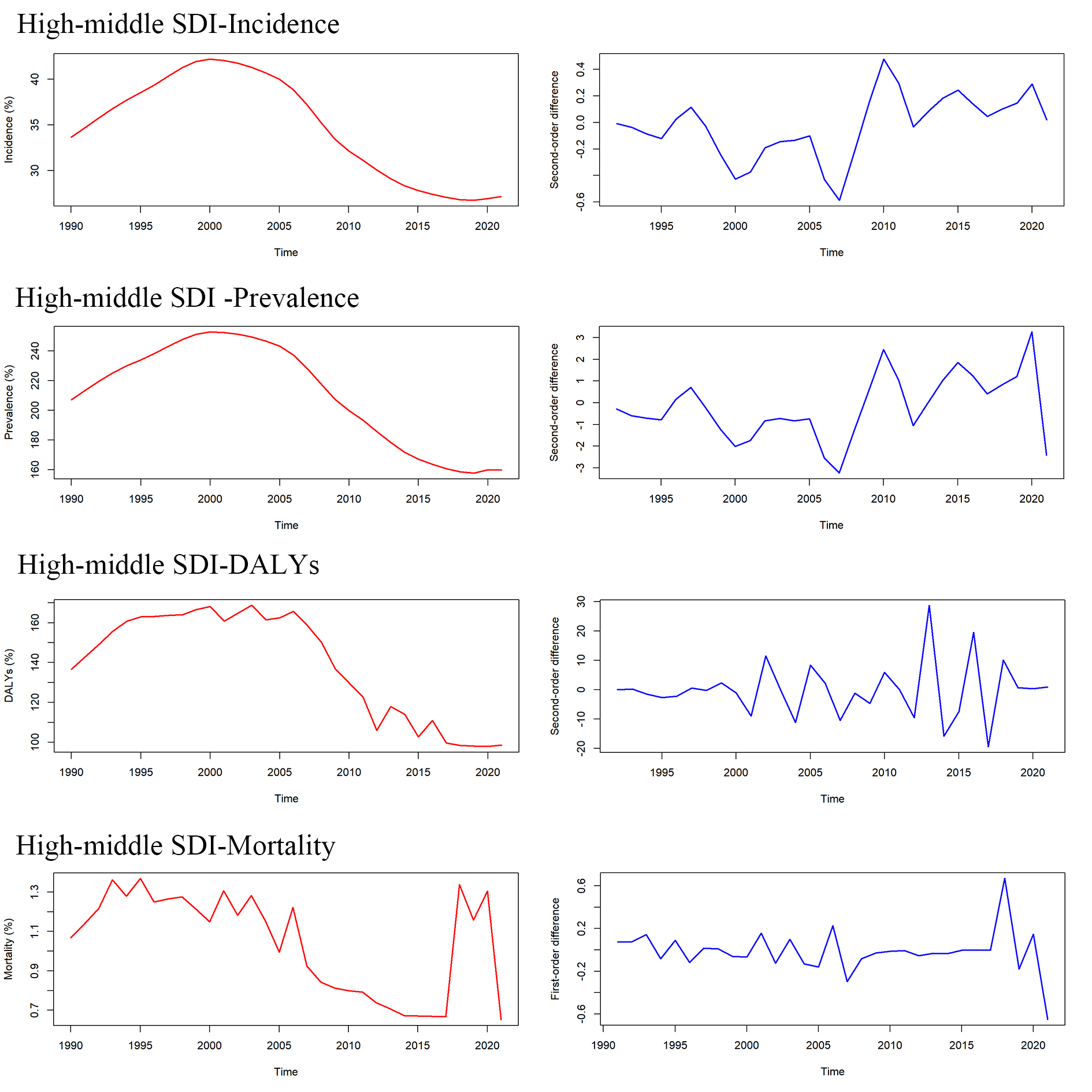


Supplementary figure 18 Timing diagram of opioid use disorder (OUD) incidence in high-middle SDI region, prevalence,DALYs and mortality (red lines: OUD incidence, prevalence, DALYs and mortality rate; blue lines: OUD incidence, prevalence, DALYs and mortality after differencing)


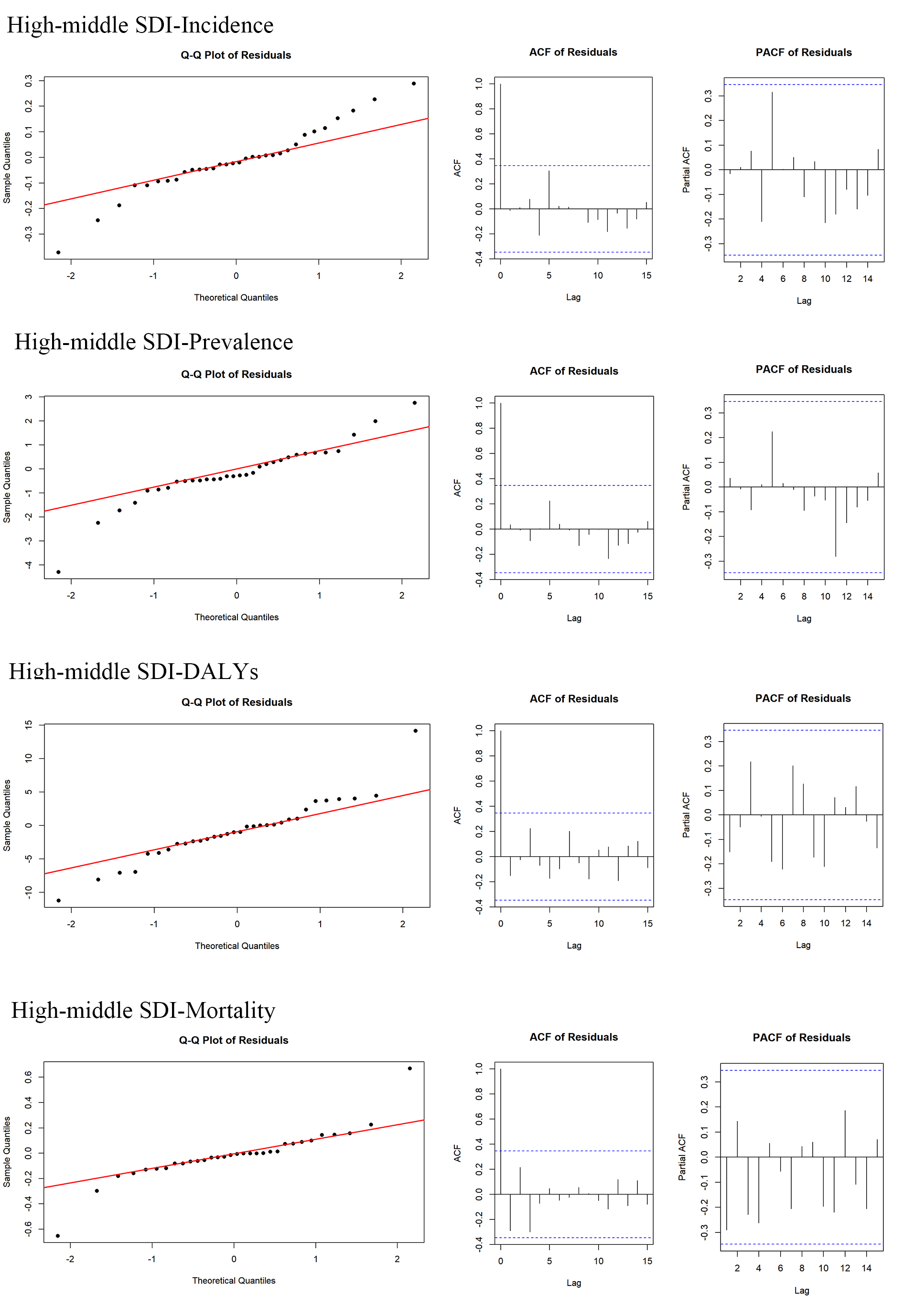


Supplementary figure 19 Residual Q-Q plots, autocorrelation function and partial autocorrelation graphs of the ARIMA models for high-middle SDI region


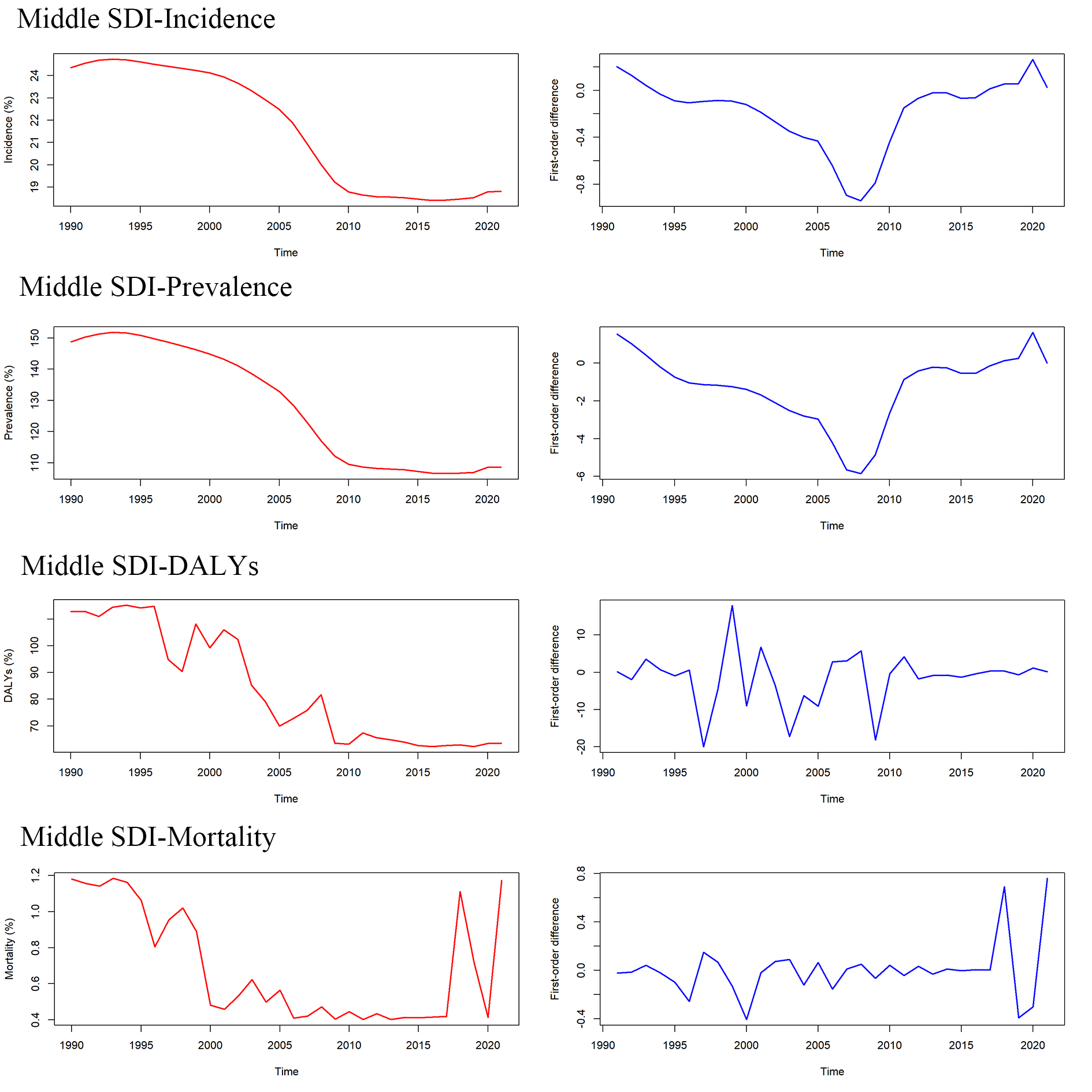


Supplementary figure 20 Timing diagram of opioid use disorder (OUD) incidence in Middle SDI region, prevalence,DALYs and mortality (red lines: OUD incidence, prevalence, DALYs and mortality rate; blue lines: OUD incidence, prevalence, DALYs and mortality after differencing)


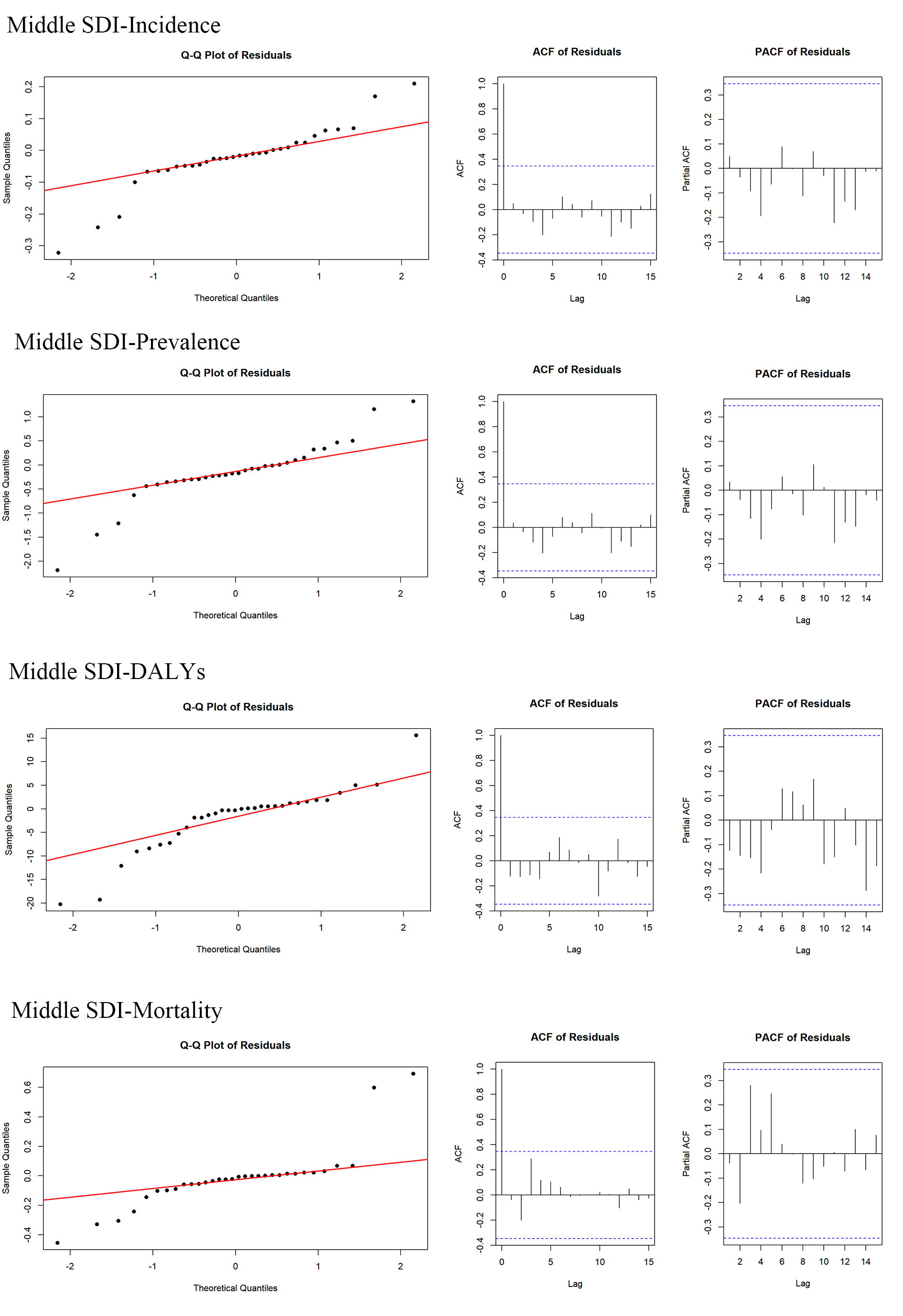


Supplementary figure 21 Residual Q-Q plots, autocorrelation function and partial autocorrelation graphs of the ARIMA models for Middle SDI region


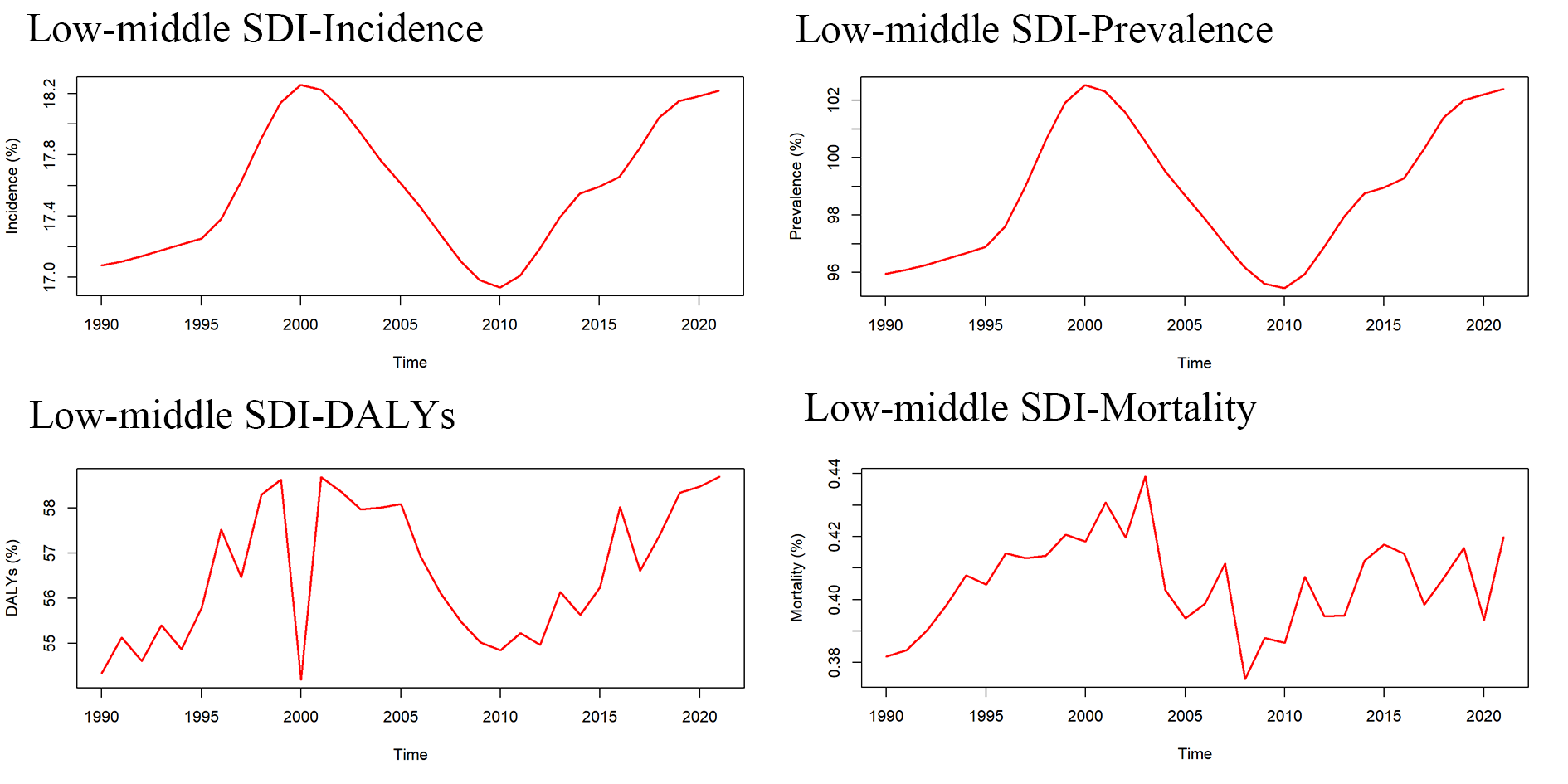


Supplementary figure 22 Timing diagram of opioid use disorder (OUD) incidence in low-middle SDI region, prevalence,DALYs and mortality


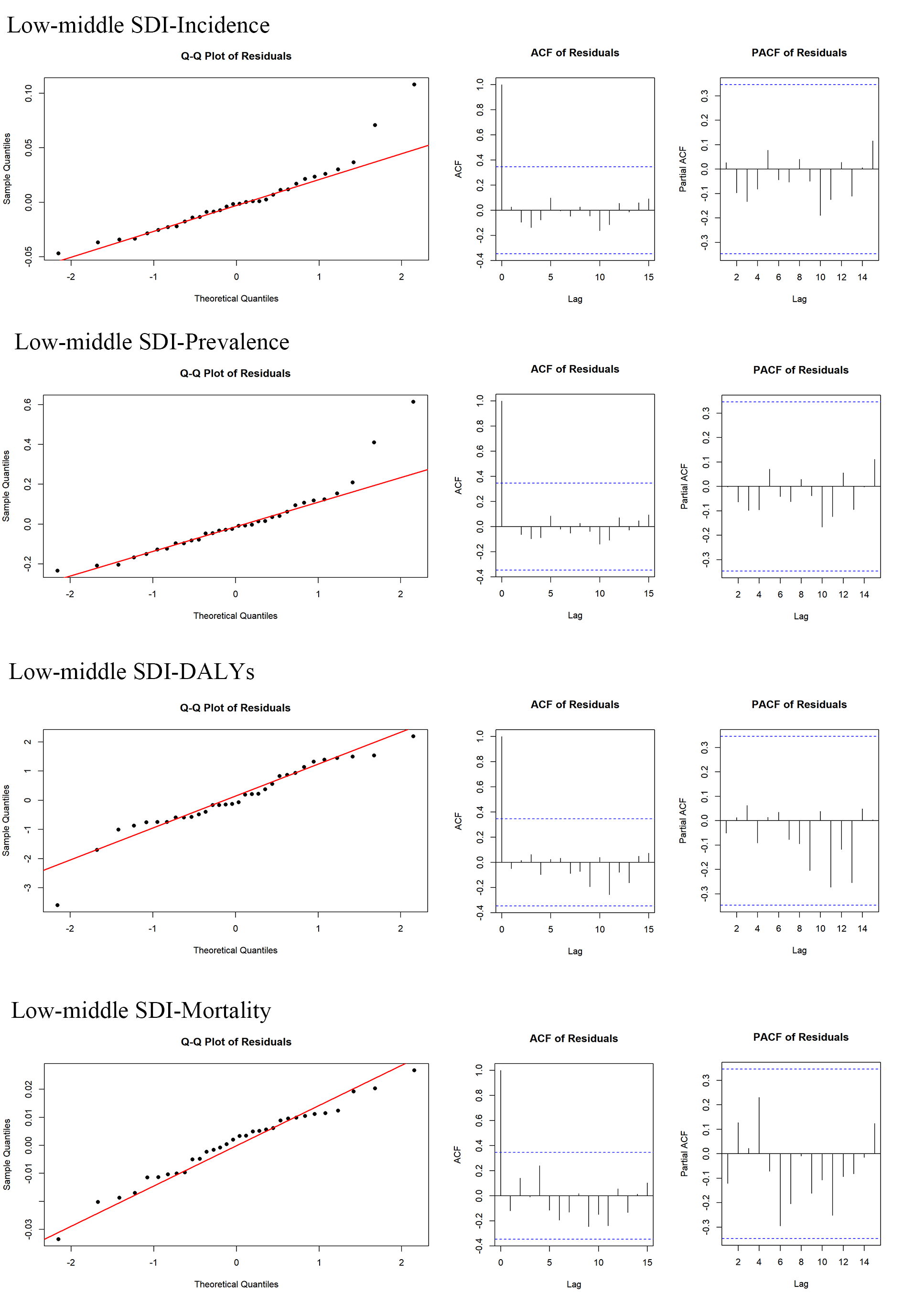


Supplementary figure 23 Residual Q-Q plots, autocorrelation function and partial autocorrelation graphs of the ARIMA models for low-middle SDI region


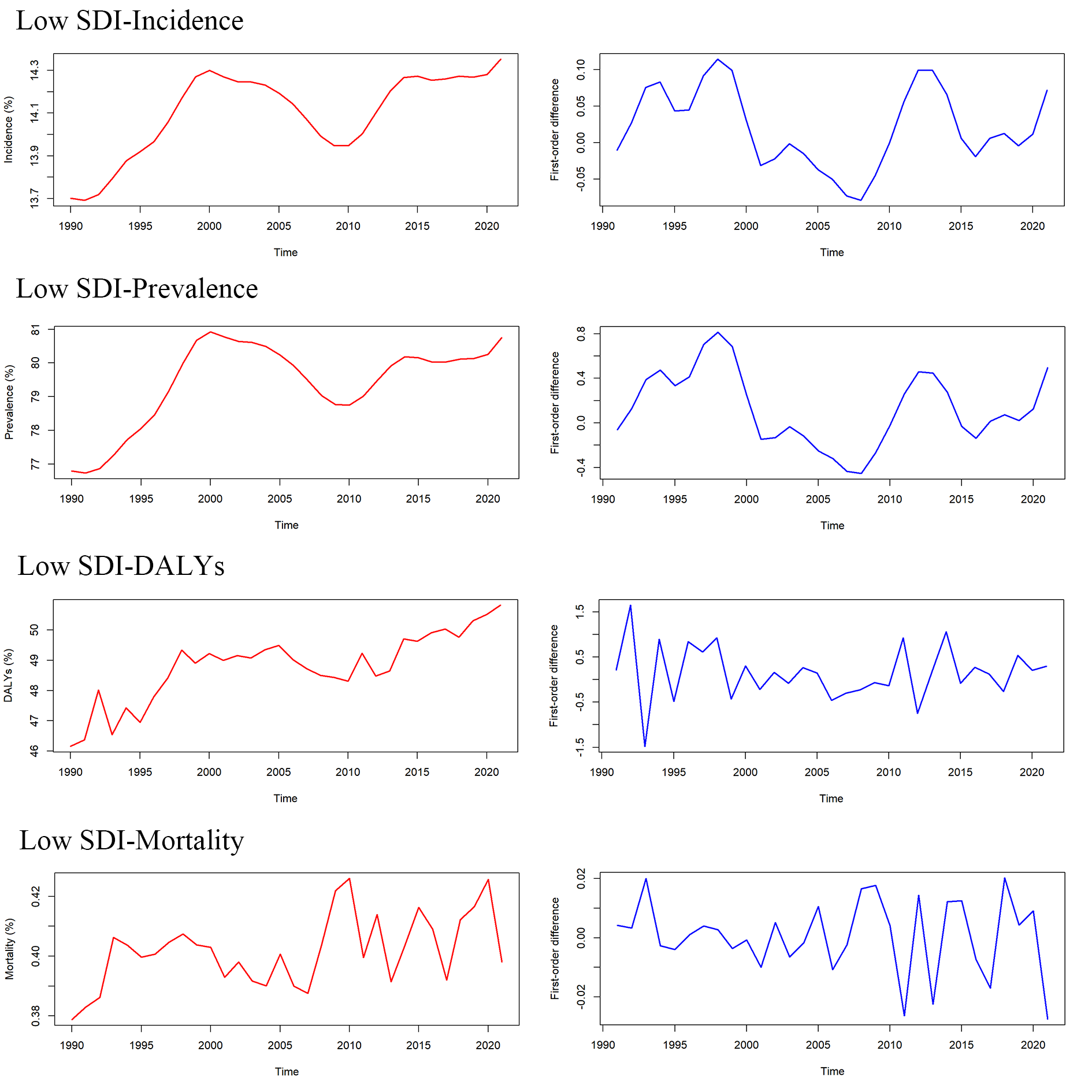


Supplementary figure 24 Timing diagram of opioid use disorder (OUD) incidence in low SDI region, prevalence,DALYs and mortality (red lines: OUD incidence, prevalence, DALYs and mortality rate; blue lines: OUD incidence, prevalence, DALYs and mortality after differencing)


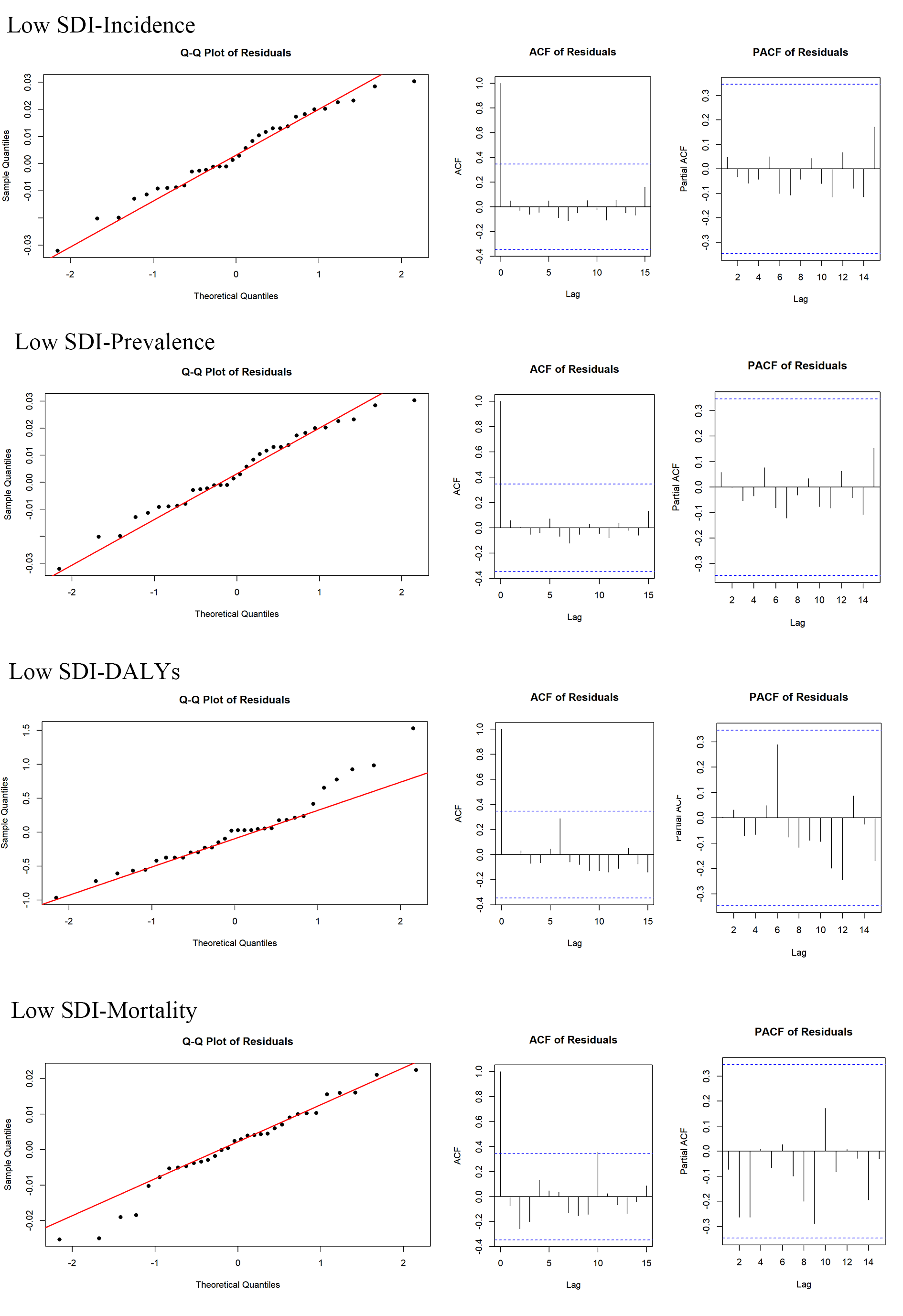


Supplementary figure 25 Residual Q-Q plots, autocorrelation function and partial autocorrelation graphs of the ARIMA models for low SDI region
